# Nonalcoholic Fatty Liver Disease as a Risk Marker for Incident Type 2 Diabetes Mellitus: A Systematic Review

**DOI:** 10.7759/cureus.113744

**Published:** 2026-07-31

**Authors:** Mudathir Elyas Suleiman Khamees, Egbal Abdalrahman Mohamed Ali, Esra Bushra Hassan Babiker, Rayan Saeed, Elkhansaa A Elsamani, Fatima Abdelmoneim Yousif Mohamed, Asim Ahmed

**Affiliations:** 1 Internal Medicine, Faculty of Medicine and Surgery, Alzaiem Alazhari University, Khartoum North, SDN; 2 Internal Medicine, Prince Sultan Armed Forces Hospital, Madinah, SAU; 3 Internal Medicine, Mahayel General Hospital, Aseer, SAU; 4 General Medicine, The National Ribat University, Khartoum, SDN; 5 Internal Medicine, University Hospital Galway, Galway, IRL; 6 Internal Medicine, King Fahad Armed Forces Hospital, Jeddah, SAU; 7 Medicne and Surgery, University of Gezira, Wad Madani, SDN

**Keywords:** incident diabetes, longitudinal studies, non-alcoholic fatty liver disease, systematic review, type 2 diabetes mellitus

## Abstract

Nonalcoholic fatty liver disease (NAFLD), now largely encompassed by the newer metabolic dysfunction-associated steatotic liver disease (MASLD) nomenclature, is increasingly recognized as a marker of systemic metabolic dysfunction. This systematic review synthesized longitudinal evidence from adults examining NAFLD and related steatotic liver disease definitions in relation to incident type 2 diabetes mellitus. PubMed/MEDLINE and Scopus were searched from inception, the supplementary search was repeated to capture recently published studies. Eligible longitudinal reports excluded participants with diabetes at baseline or clearly identified new-onset diabetes during follow-up. Because the included cohorts differed in liver disease definitions, diabetes ascertainment, populations, follow-up structures, and reported effect measures, the findings were synthesized narratively. The protocol was prepared before screening but was not prospectively registered. A total of 61 reports were included. Risk of bias was generally low to moderate where adequately assessed, while reports with incomplete domain-level information were interpreted cautiously. NAFLD or MASLD was generally associated with higher estimates of incident diabetes risk, although the magnitude varied and was attenuated after adjustment for metabolic factors in some studies. Persistent or newly developed steatosis and a greater liver fat or fibrosis burden were associated with higher estimates of future diabetes occurrence, whereas the resolution or reduction of liver fat was associated with lower estimates. Reported subgroup differences arose largely from individual or overlapping cohorts and were not sufficiently consistent to establish uniform effect modification. Overall, the evidence supports NAFLD or MASLD as a clinical risk marker rather than a stand-alone individualized prediction tool and supports standardized longitudinal assessments in future studies.

## Introduction and background

Type 2 diabetes mellitus (T2DM) is a major global public health problem because of its increasing prevalence, long-term vascular and metabolic complications, and substantial healthcare burden [1,2]. Established risk factors include obesity, family history, and impaired glycemic regulation. Because non-alcoholic fatty liver disease (NAFLD) reflects several metabolic abnormalities that may be detectable before overt diabetes develops, it may provide additional clinical information about the future occurrence of diabetes [1,2].

NAFLD, now increasingly discussed within the broader framework of metabolic dysfunction-associated steatotic liver disease (MASLD), is among the most common chronic liver diseases worldwide and is closely associated with obesity, insulin resistance, dyslipidemia, and hypertension [3-7]. Most earlier longitudinal studies used NAFLD definitions, whereas newer reports may apply metabolic dysfunction-associated fatty liver disease (MAFLD) or MASLD criteria. In this review, studies were considered according to their prespecified steatotic liver disease definitions; these terms were not assumed to be fully interchangeable, and definitional differences were considered during synthesis and interpretation. Beyond its hepatic manifestations, NAFLD represents a marker of systemic metabolic dysfunction. Hepatic steatosis is linked to insulin resistance, chronic low-grade inflammation, and altered glucose and lipid metabolism, providing biological plausibility for an association with subsequent T2DM [8-11].

Previous longitudinal studies and meta-analyses have demonstrated an association between NAFLD and incident T2DM [8,9,12,13]. However, earlier syntheses primarily focused on the overall association, while important uncertainties remain regarding dynamic liver disease status, the resolution or progression of steatosis, liver fat and fibrosis burden, population subgroups, differences in exposure and outcome definitions, and variation in confounder adjustment. Some metabolic variables may represent confounders, intermediates within the causal pathway, or both; therefore, the extent to which the association persists after metabolic adjustment remains uncertain. Updated evidence also requires consideration of methodological quality and reports arising from overlapping cohort datasets.

Accordingly, the primary review question was whether NAFLD or a related prespecified steatotic liver disease definition, compared with the absence of the corresponding condition or with alternative disease status categories, is associated with incident T2DM among adults without diabetes at baseline. Secondary aims were to examine variation according to diagnostic approach, dynamic disease status, liver disease severity, population characteristics, reported effect measures, and risk of bias.

## Review

Methods

Review Design

This systematic review was reported in accordance with the Preferred Reporting Items for Systematic Reviews and Meta-Analyses (PRISMA) 2020 statement [14]. The completed PRISMA 2020 checklist is provided in Appendix 1. The protocol was prepared before screening and data extraction but was not prospectively registered; retrospective registration was not pursued after the review activities had commenced. No separate amendment log was retained. A narrative synthesis was planned because the eligible reports addressed related but non-interchangeable clinical questions and used heterogeneous populations, exposure definitions, outcome methods, comparator groups, follow-up structures, covariate adjustment, and effect measures.

Information Sources and Search Strategy

The original systematic search covered PubMed/MEDLINE and Scopus from database inception through December 31, 2022. The retained original screening log contained a combined total of 722 records but did not preserve database-specific yields from the original search or the exact execution date. To correct the supplementary update, the search was repeated for records published from January 1, 2023 through July 22, 2026. The repeated search identified 77 PubMed/MEDLINE records and 120 Scopus records before cross-database deduplication. Seven duplicate records were removed, leaving 190 unique records for screening. No other bibliographic databases were searched.

No language restriction was applied at the search stage. Studies were included only when sufficient English-language information was available for eligibility assessment and data extraction. Eligible sources were published, peer-reviewed reports. Unpublished studies, dissertations, theses, grey literature, animal studies, in vitro studies, and conference abstracts without sufficient extractable data were excluded.

The corrected supplementary search used pragmatic concepts for steatotic liver disease and incident or new-onset diabetes. In PubMed/MEDLINE, title/abstract terms covered NAFLD, MAFLD, MASLD, non-alcoholic or nonalcoholic fatty liver disease, and metabolic dysfunction-associated or metabolic-associated steatotic/fatty liver disease, combined with terms for incident or new-onset diabetes, diabetes development, or diabetes risk; the publication-date field restricted the results to the supplementary period. Scopus records were retrieved through the review team’s Scopus-connected search interface using a natural-language question specifying longitudinal adult studies, NAFLD/MASLD/MAFLD, incident type 2 diabetes, the absence of diabetes at baseline, and the same publication period. The exact July 22, 2026 cutoff was applied during screening. Reference lists and previously eligible reports from the update period were also rechecked. Three previously included eligible reports from the supplementary period were not re-retrieved by the repeated database query but were retained after their eligibility was reconfirmed. The documented search details are presented in Table 1.

**Table 1:** Documented database-specific free-text search strategies and supplementary update details. The search strings are reproduced as documented. The original records did not preserve complete line-by-line database search histories or database-specific yields from the original search. The phrase “metabolic-associated fatty liver disease” is retained in the displayed historical search string because it reflects the documented query; throughout the narrative, MAFLD is expanded as metabolic dysfunction-associated fatty liver disease. MASLD: Metabolic dysfunction-associated steatotic liver disease; MEDLINE: Medical Literature Analysis and Retrieval System Online; NAFLD: Nonalcoholic fatty liver disease; T2DM: Type 2 diabetes mellitus; TITLE-ABS-KEY: Title, abstract, and keywords field.

Database	Documented search string and update details
PubMed/MEDLINE	("nonalcoholic fatty liver disease"[Title/Abstract] OR "non-alcoholic fatty liver disease"[Title/Abstract] OR NAFLD[Title/Abstract] OR "metabolic dysfunction-associated steatotic liver disease"[Title/Abstract] OR "metabolic dysfunction associated steatotic liver disease"[Title/Abstract] OR MASLD[Title/Abstract] OR "metabolic-associated fatty liver disease"[Title/Abstract] OR "metabolic associated fatty liver disease"[Title/Abstract] OR MAFLD[Title/Abstract]) AND ("incident type 2 diabetes"[Title/Abstract] OR "incident type 2 diabetes mellitus"[Title/Abstract] OR "incident diabetes"[Title/Abstract] OR "new-onset type 2 diabetes"[Title/Abstract] OR "new onset type 2 diabetes"[Title/Abstract] OR "new-onset diabetes"[Title/Abstract] OR "new onset diabetes"[Title/Abstract] OR "development of type 2 diabetes"[Title/Abstract] OR "development of diabetes"[Title/Abstract] OR "risk of type 2 diabetes"[Title/Abstract] OR "risk of diabetes"[Title/Abstract]) AND ("2023/01/01"[Date - Publication] : "2026/07/22"[Date - Publication]). Corrected supplementary search: n = 77 before cross-database deduplication.
Scopus	Research question entered in the Scopus-connected search interface: “What longitudinal studies published between January 1, 2023 and July 22, 2026 evaluated non-alcoholic fatty liver disease (NAFLD), metabolic dysfunction-associated steatotic liver disease (MASLD), or metabolic-associated fatty liver disease (MAFLD) as a risk marker for incident type 2 diabetes mellitus in adults without diabetes at baseline?” Corrected supplementary search: n = 120 before cross-database deduplication.

Eligibility Criteria

Studies were selected according to predefined eligibility criteria structured using the Population, Exposure, Comparator, and Outcome (PECO) framework. The inclusion and exclusion criteria are presented in Table 2, with each criterion listed in a separate row.

**Table 2:** PECO framework and eligible study designs. This table presents the predefined inclusion and exclusion criteria used for study selection. MAFLD: Metabolic dysfunction-associated fatty liver disease; MASLD: Metabolic dysfunction-associated steatotic liver disease; NAFLD: Nonalcoholic fatty liver disease; PECO: Population, Exposure, Comparator, and Outcome; T2DM: Type 2 diabetes mellitus.

Domain	Inclusion criteria	Exclusion criteria
Population	Adults aged 18 years or older without T2DM at baseline, or adult cohorts in which new-onset T2DM was clearly identified during follow-up.	Pediatric or pregnant populations without separable adult data; cohorts in which incident diabetes could not be distinguished from diabetes already present at baseline.
Steatotic liver exposure	NAFLD, MAFLD, MASLD, or another prespecified steatotic liver disease definition assessed using imaging, histology, a validated noninvasive index, quantitative liver fat measurement, or a clearly described surrogate definition.	Liver exposure not clearly defined; studies focused primarily on viral hepatitis, alcohol-related liver disease, or another chronic liver disease without extractable steatotic liver disease findings.
Comparator/contrast	Absence of the corresponding liver condition, alternative disease status or severity categories, BMI-defined groups, or clinically relevant within-disease categories.	No interpretable exposure contrast and no eligible within-disease comparison relevant to incident T2DM.
Outcome	Incident T2DM during longitudinal follow-up according to the definition used by the original cohort.	Only prevalent diabetes reported; type 1 diabetes only; or no longitudinal incident T2DM outcome.
Temporality/follow-up	Baseline or time-varying steatotic liver disease status linked prospectively or retrospectively to subsequent incident T2DM during follow-up.	Cross-sectional assessment only or no temporal link between liver status and subsequent diabetes occurrence.
Study design	Prospective or retrospective cohort studies, health-check or occupational cohorts, registry or database cohorts, and clinical longitudinal follow-up studies.	Cross-sectional studies without longitudinal follow-up; case reports; non-original publications.
Publication type	Published peer-reviewed reports with sufficient information for eligibility assessment and data extraction.	Reviews, systematic reviews, meta-analyses, editorials, letters, commentaries, dissertations, theses, animal studies, in vitro studies, and conference abstracts without sufficient extractable data.
Language/information sufficiency	No language restriction at the search stage; sufficient English-language information available for eligibility assessment and extraction.	Insufficient accessible information to establish eligibility or extract the required longitudinal data.

Studies were eligible when incident T2DM could be linked longitudinally to a baseline or time-varying steatotic liver disease definition in adults without diabetes at baseline. A report could contribute to more than one analytical theme when it reported several relevant comparisons.

Studies were excluded if they were cross-sectional only, reported prevalent diabetes without a longitudinal assessment of incident diabetes, did not clearly define the liver exposure, focused only on type 1 diabetes, included pediatric or pregnant populations without separable adult data, or primarily involved viral hepatitis, alcohol-related liver disease, or other chronic liver diseases without extractable steatotic liver disease findings. Reviews, systematic reviews, meta-analyses, editorials, letters, commentaries, animal studies, in vitro studies, dissertations, theses, and conference abstracts without sufficient data were excluded.

Study Selection

Microsoft Excel was used to manage and document screening. In the corrected supplementary search, duplicate records were identified first by DOI and then manually verified using normalized titles, first-author names, and publication years. A.A. and M.E.S.K. independently assessed the titles and abstracts and then the retrieved full-text reports against the predefined eligibility criteria. Records were not divided between reviewers. No automation tools were used for eligibility decisions, and the reviewers were not blinded to authors, institutions, or journal names. Disagreements were resolved through discussion and consensus. Formal inter-reviewer agreement using kappa was not calculated.

The specific reason for exclusion was recorded for each excluded full-text report for which a screening record was available. Reports from potentially overlapping cohorts were identified using cohort names, institutions, recruitment periods, participant descriptions, sample sizes, author groups, and data sources. Related reports, particularly those from NAGALA and the Kangbuk Samsung Health Study, were not interpreted as independent replications of the same association. The identified overlapping cohorts and related reports are mapped in Appendix 3.

Data Extraction and Management of Incomplete Information

A.A. and M.E.S.K. independently extracted data using a standardized Microsoft Excel form that was piloted on five included studies. Extracted information included study and population characteristics; liver disease definition and assessment; comparator and baseline diabetes status; follow-up; incident T2DM definition and event numbers; crude and adjusted effect estimates; adjustment variables; subgroup and sensitivity analyses; cohort overlap; and key methodological limitations. Discrepancies were resolved through discussion and consensus.

When several models were reported, the multivariable model identified by the original investigators as the principal analysis was prioritized. If no principal model was specified, the most comprehensively adjusted model was summarized. Crude or less-adjusted estimates were used only when an adjusted estimate was unavailable or when comparisons across models were necessary to demonstrate attenuation. Study authors were not contacted for missing information. Information unavailable in the retained review materials was marked as not reported or unclear rather than reconstructed, and incompletely reported studies were not used alone to support major numerical conclusions.

Outcomes and Analytical Themes

The primary outcome was incident T2DM during longitudinal follow-up according to the definition used in each original cohort. Accepted definitions included laboratory criteria, repeated health-check data, physician diagnosis, medication use, claims data, diagnostic coding, registry linkage, or database-defined new-onset diabetes.

Analytical themes included disease persistence, development, or resolution; liver fat burden, steatosis grade, and fibrosis stage; obesity-status phenotypes; sex and menopausal differences; age-group and racial differences; lifestyle modifiers; prior gestational diabetes; and prediction-focused findings.

Data Synthesis and Assessment of Quantitative Pooling

The feasibility of quantitative pooling was reassessed after extraction by grouping reports according to exposure method, comparator, population, effect measure, and cohort independence. Apparently similar studies remained clinically and analytically heterogeneous: USG-based cohorts included general, occupational, health-check, lean, non-obese, prediabetes, and special populations; index-based exposures embedded metabolic variables; biopsy cohorts evaluated different histologic dimensions; and several reports arose from overlapping datasets. HRs, RRs, ORs, incidence rates, prediction metrics, and formal interaction results addressed different estimands and were not treated as interchangeable. A random-effects meta-analysis was therefore not performed because a pooled value would imply comparability that the evidence did not support.

Findings were synthesized using principles from the Synthesis Without Meta-analysis reporting guideline [15]. Studies were grouped by clinical question: overall etiological association; dynamic disease status; steatosis, liver fat, or fibrosis severity; BMI-defined and other population subgroups; clinical or lifestyle modifiers; and prediction-focused findings. Within each group, the direction, approximate magnitude, precision, diagnostic approach, outcome ascertainment, follow-up, adjustment set, and risk of bias were compared descriptively. No statistical heterogeneity threshold or test was applied because no pooled analysis was performed. Structured effect-estimate tables and forest-style plots without pooled estimates are provided in Appendix 6; HRs, RRs, and ORs are displayed separately.

Risk of Bias Assessment

Observational cohort reports were appraised using the Newcastle-Ottawa Scale (NOS) [16], which covers the selection, comparability, and outcome domains. Review-specific thresholds classified 7-9 stars as low risk, 5-6 stars as moderate risk, and fewer than 5 stars as high risk. The same domain framework was applied to reports identified in the corrected supplementary search. For older reports in which complete domain-level information could not be reconstructed from the retained materials, unavailable domains were marked as unclear rather than inferred. Prediction-model findings were treated as secondary and were not used as etiological evidence.

A.A. and M.E.S.K. independently performed the available risk-of-bias assessments, and disagreements were resolved by consensus. Risk of bias did not determine eligibility, but reports with greater concerns or incomplete domain-level information were not used alone to support the principal conclusions. Study-level appraisal information is presented in Appendix 4, and the domain-level traffic-light display is shown in Appendix 8.

Certainty of Evidence, Reporting Bias, and Missing Results

A formal GRADE rating was not performed. This decision was not based on the use of narrative synthesis; instead, confidence in each major conclusion was evaluated in a structured qualitative table considering risk of bias, consistency, directness, precision, and possible publication bias. These judgments are not presented as formal GRADE categories. Publication bias was not assessed using funnel plots or statistical tests for small-study effects because there was no sufficiently comparable pooled set. Selective publication and selective reporting remained possible, particularly for small studies, subgroup analyses, prediction models, data-driven thresholds, and positive findings, and were considered when interpreting the evidence. The structured qualitative confidence judgments are presented in Appendix 5.

Ethical Considerations

Ethical approval was not required because this systematic review used data from previously published studies and did not involve individual participant data.

Results

Study Selection

**Figure 1:**
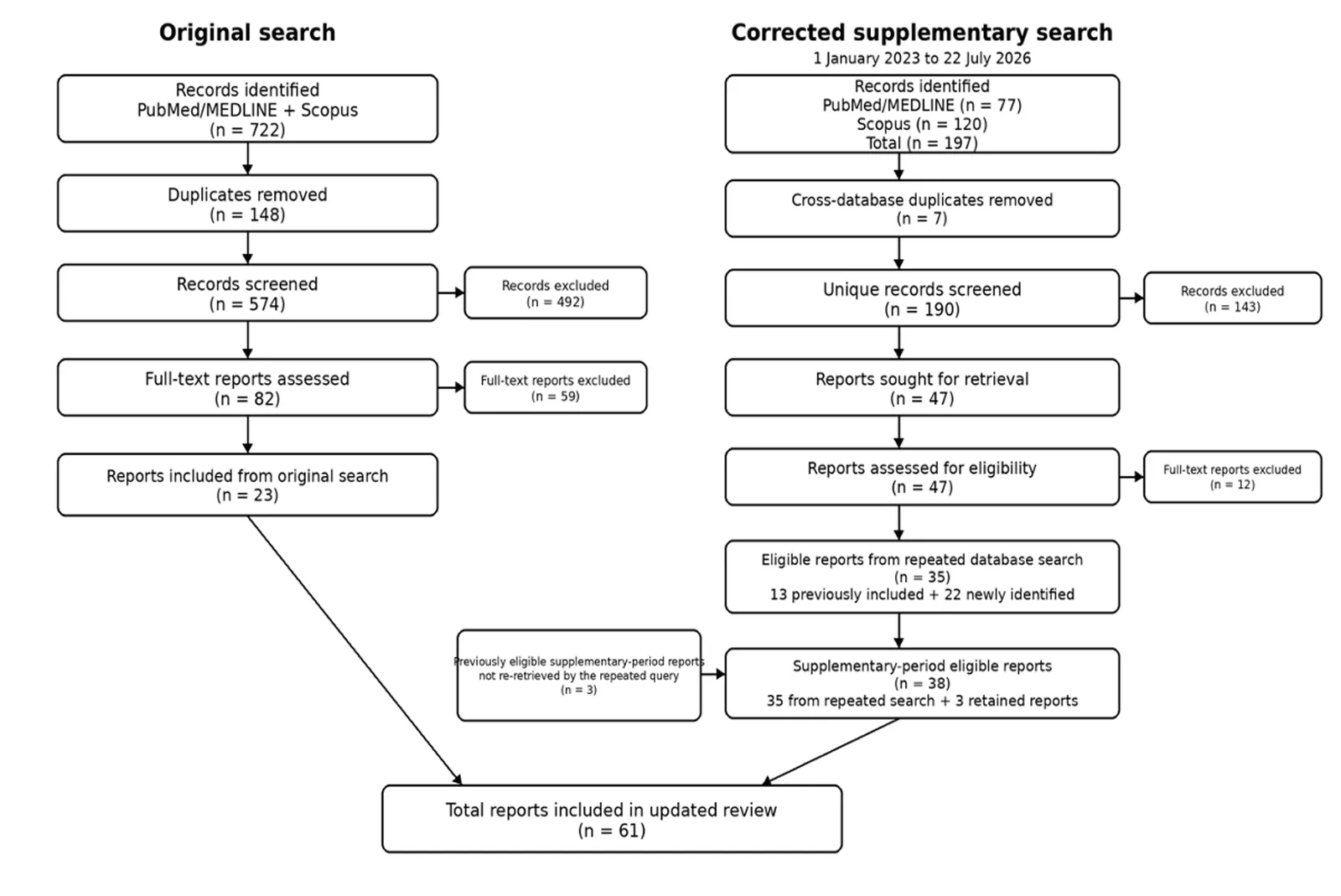
PRISMA 2020 flow diagram of study identification, screening, eligibility assessment, and inclusion. PRISMA 2020 flow diagram of study selection for the original search and corrected supplementary search. The original search yielded 722 records and 23 eligible reports. The corrected supplementary database search yielded 197 records, including 190 unique records after cross-database deduplication, and 35 eligible reports after full-text assessment; 13 of these were already present in the submitted update, and 22 were newly identified. Three previously eligible reports from the supplementary period that were not re-retrieved by the repeated query were retained after their eligibility was reconfirmed. The final systematic review therefore included 61 reports. PRISMA: Preferred Reporting Items for Systematic Reviews and Meta-Analyses; T2DM: Type 2 diabetes mellitus.

The original database search identified 722 records. After removal of 148 duplicates, 574 records were screened by title and abstract, of which 492 were excluded. Eighty-two full-text reports were assessed, and 59 were excluded, leaving 23 longitudinal reports in the original qualitative synthesis [17-39]. The corrected supplementary PubMed/MEDLINE and Scopus search identified 197 records: 77 from PubMed/MEDLINE and 120 from Scopus. After removal of seven cross-database duplicates, 190 unique records were screened, and 143 were excluded during title and abstract screening. Forty-seven full-text reports were assessed; 12 were excluded, leaving 35 eligible reports in the repeated search. Thirteen of these had already been included in the submitted supplementary evidence set, while 22 were newly identified. Three additional eligible reports from the supplementary period had been included previously but were not re-retrieved by the repeated query; their eligibility was reconfirmed, and they were retained. The supplementary period therefore contributed 38 eligible reports, and the updated review comprised 61 reports in total. Full-text exclusion reasons from the corrected supplementary search are summarized in Appendix 7. The study selection process is summarized in Figure 1.

Study Characteristics

The synthesis included 61 longitudinal reports [17-77], comprising prospective and retrospective cohorts, health-check and occupational cohorts, population databases, clinical and biopsy-confirmed cohorts, and secondary analyses of established datasets. The evidence spanned multiple regions and included very large national datasets as well as smaller clinical cohorts. Liver disease was assessed using USG, computed tomography, controlled attenuation parameter, fatty liver index (FLI) and other noninvasive indices, biopsy or histology, quantitative liver fat measurement, and clearly described surrogate definitions. Several reports arose from overlapping datasets, particularly NAGALA, Korean national health-screening data, Samsung/Kangbuk health-check cohorts, ELSA-Brasil, and Taiwanese health-check cohorts, and were interpreted as related reports rather than independent replications. The characteristics of all included reports are summarized in Table 3.

**Table 3:** Characteristics of included longitudinal reports evaluating the association of NAFLD, MAFLD, or MASLD with incident T2DM. This table summarizes the study design, population, follow-up, liver disease assessment, incident T2DM ascertainment, and principal findings. Risk-of-bias information is reported separately in Appendix 4. Several newer reports are secondary analyses of overlapping cohorts and should not be interpreted as independent replications. ALT: Alanine aminotransferase; APRI: Aspartate aminotransferase-to-platelet ratio index; CAP: Controlled attenuation parameter; FLI: Fatty liver index; GDM: Gestational diabetes mellitus; HbA1c: Glycated hemoglobin; HSI: Hepatic steatosis index; MAFLD: Metabolic dysfunction-associated fatty liver disease; MASLD: Metabolic dysfunction-associated steatotic liver disease; NAGALA: NAFLD in the Gifu Area, Longitudinal Analysis; NAFLD: Nonalcoholic fatty liver disease; NOS: Newcastle-Ottawa Scale; OGTT: Oral glucose tolerance test; RR: Relative risk; T2DM: Type 2 diabetes mellitus.

Study and citation	Setting, design, and population	Follow-up and liver assessment	Incident T2DM ascertainment	Principal finding
Adams LA et al., 2009 [17]	Australia; Busselton Health Survey; comparative longitudinal cohort; adult residents; 109 with ALT-defined NAFLD and 249 without NAFLD; n = 358	11.1 years; elevated ALT >40 IU/L after exclusion of alcohol-related, viral, metabolic, and autoimmune liver disease; surrogate rather than imaging-confirmed steatosis	Diabetes status reassessed at follow-up; exact diagnostic criteria not stated in the abstract	Higher crude incidence; NAFLD was no longer statistically independent after adjustment (adjusted estimate not reported in the abstract). Events: 20/106 (18.9%) versus 15/246 (6.1%) after excluding baseline diabetes
Cai X et al., 2022 [18]	Japan; NAGALA cohort; longitudinal observational prediction-model study; obese participants with NAFLD; n = 1,820	Not reported in the PubMed abstract; NAFLD definition not reported in the PubMed abstract	Incident T2DM prediction endpoint; exact criteria not reported in the abstract	Five predictors selected from 17 variables; AUCs and the C-index indicated predictive performance, but numerical values were not reported in the abstract. Events: not reported in the PubMed abstract
Cho HJ et al., 2019 [19]	Republic of Korea; routine healthcare assessment cohort; prospective health-check cohort; persistent NAFLD, 670; resolved NAFLD, 155; incident NAFLD, 498; no NAFLD, 1,403; n = 2,726	Serial follow-up; exact duration not reported in the PubMed abstract; serial abdominal ultrasonography; fatty liver index used as a sensitivity or replication measure	Incident diabetes during follow-up; exact criteria not stated in the abstract	Persistent NAFLD: HR 3.59 (95% CI 2.05-6.27); incident NAFLD: HR 1.94 (1.08-3.50); resolved NAFLD was not significantly different from no NAFLD. Events: not reported in the PubMed abstract
Chung GE et al., 2021 [20]	Republic of Korea; Korean National Health Insurance Service; nationwide population-based cohort; adults aged 20-39 years who underwent a health examination; n = 5,254,786	Median 8.6 years; fatty liver index ≥60; alcohol consumption ≥30 g/day excluded	Newly diagnosed diabetes identified using claims data	Adjusted HR 4.97 (95% CI 4.90-5.05). Events: 91,885 incident diabetes cases
Fan JG et al., 2007 [21]	China; Bao-Steel employee cohort; occupational longitudinal matched cohort; 358 participants with fatty liver and 788 age-, sex-, and occupation-matched controls; n = 1,146	Median 6 years; fatty liver identified during biennial medical checkups; exact imaging criteria not stated in the abstract	Diabetes identified during follow-up medical checkups; exact criteria not stated in the abstract	Incidence comparisons only; no adjusted association estimate reported in the abstract. Events: diabetes incidence of 20.3% versus 5.2%; non-obese subgroup, 11.1% versus 4.3%
Fukuda T et al., 2016 [22]	Japan; long-term health-check program; population-based retrospective health-check cohort; overweight and non-overweight adults with and without NAFLD; n = 4,629	Mean 12.8 years; standardized questionnaire and abdominal ultrasonography	Incident T2DM as the primary outcome; exact criteria not stated in the abstract	Compared with non-overweight participants without NAFLD: HR 3.59 (95% CI 2.14-5.76) for non-overweight participants with NAFLD; 1.99 (1.47-2.69) for overweight participants without NAFLD; and 6.77 (5.17-8.91) for overweight participants with NAFLD. Events: 351 incident T2DM cases
Kogiso T et al., 2021 [23]	Clinical biopsy-confirmed NAFLD cohort; 272 participants without diabetes at baseline included in the incident-diabetes analysis (full cohort, n = 544)	10-year incidence reported; liver biopsy	De novo diabetes and diabetes-treatment course; exact diagnostic criteria not stated in the abstract	Higher incidence among patients with obesity and the KCNQ1 CC genotype; numerical adjusted association not reported in the abstract. Events: 44 de novo diabetes cases; 10-year incidence, 17.9%
Kim Y et al., 2022 [24]	Republic of Korea; adult health-screening cohort; large longitudinal cohort; 109,810 premenopausal women, 4,958 postmenopausal women, and 130,286 men without diabetes; n = 245,054	Median 5.3 years; NAFLD diagnostic method not stated in the PubMed abstract; severity assessed using serum fibrosis markers	Incident T2DM during follow-up; exact diagnostic criteria not stated in the abstract	Adjusted HR 4.63 (95% CI 4.17-5.14) in premenopausal women, 2.65 (2.02-3.48) in postmenopausal women, and 2.16 (2.04-2.29) in men. Events: 8,381 incident T2DM cases
Dai W et al., 2021 [25]	China; Second Xiangya Hospital cohort; cohort study with a cross-sectional baseline and longitudinal follow-up; 16,093 non-obese adults at baseline; 12,649 free of T2DM and CAD entered follow-up; n = 12,649	5 years; ultrasonography; non-obese defined as BMI <25 kg/m²	Incident T2DM during follow-up; exact diagnostic criteria not stated in the abstract	Adjusted HR 2.3 (95% CI 1.7-3.2). Events: 177 incident T2DM cases
Tang X et al., 2022 [26]	China; Ningbo company-employee cohort; retrospective occupational cohort; company employees followed between 2006 and 2016; n = 13,032	11 years; NAFLD identified through occupational health examinations; exact diagnostic criteria not stated in the abstract	Development of diabetes during follow-up; exact criteria not stated in the abstract	Descriptive outcome proportions; no adjusted NAFLD-versus-control diabetes estimate reported. Events: diabetes developed in 12.6% of male and 11.6% of female participants with NAFLD
Zhang H et al., 2022 [27]	United Kingdom; UK Biobank; prospective cohort; adults without T2DM at baseline; n = 365,339	Median 11.0 years; fatty liver index	Incident T2DM during follow-up; exact ascertainment algorithm not stated in the abstract	NAFLD plus a poor sleep pattern: RR 3.17 (95% CI 2.80-3.59); the overall sleep pattern did not significantly interact with NAFLD, but the interaction with insomnia was significant (P = 0.003). Events: 8,774 incident T2DM cases
Arase Y et al., 2009 [28]	Japan; clinical ultrasonography-diagnosed NAFLD cohort; retrospective within-NAFLD cohort; patients with NAFLD; n = 6,003	Mean 4.9 years; ultrasonography-diagnosed NAFLD	Fasting or casual blood samples collected during follow-up; exact diabetes threshold not stated in the abstract	Prediabetes: HR 6.39 (95% CI 5.00-8.18); GGT >109 IU/L: HR 1.60 (1.22-2.02); triglycerides >150 mg/L (source-reported unit): HR 1.28 (1.05-1.55); physical activity <60 min/week: HR 1.60 (1.25-2.00). Events: 411 incident T2DM cases
Sanyal AJ et al., 2021 [29]	United States; NASH Clinical Research Network; prospective multicenter clinical cohort; adults across the histologic NAFLD spectrum; n = 1,773	Median 4 years; liver biopsy and histologic fibrosis stage	Incident T2DM as a nonhepatic outcome; exact diagnostic criteria not stated in the abstract	Incidence-rate comparison; no T2DM-specific adjusted effect estimate reported in the abstract. Events: T2DM incidence of 7.53 versus 4.45 events per 100 person-years for F4 versus F0-F2
Li WD et al., 2015 [30]	China; 17 medical units; multicenter cohort; 4,847 sampled and 4,736 completed follow-up; n = 4,736	Median 3.85 years (4-year follow-up; 17,223 person-years); B-mode ultrasonography	Incident T2DM during follow-up; exact criteria not fully stated in the abstract	Adjusted RR 3.367 (95% CI 2.367-4.266) for NAFLD versus control. Events: 380 incident T2DM cases; cumulative incidence, 17.4% in participants with NAFLD versus 4.1% in controls
Liu C et al., 2022 [31]	Japan; Japanese population cohort; population-based cohort; 15,464 individuals, including 2,714 participants with NAFLD; n = 15,464	16,446 person-years among participants with NAFLD; abdominal ultrasonography after excluding excessive alcohol consumption and other liver diseases	Incident T2DM during follow-up; exact diagnostic criteria not stated in the abstract	Current versus never smoking: adjusted HR 2.05 (95% CI 1.43-2.94). Events: 223/2,714 participants with NAFLD developed T2DM
Nasr P et al., 2020 [32]	Sweden; biopsy-proven NAFLD cohort; long-term biopsy-confirmed clinical cohort; 129 patients, including 106 without T2DM at baseline; n = 106	Mean 23.2 years; liver biopsy with conventional histology and stereological point counting	Incident T2DM during long-term clinical follow-up; exact criteria not stated in the abstract	Adjusted HR 1.60 per steatosis grade; HR 1.03 per percentage-point increase in quantified liver fat; reduction in liver fat: HR 0.91 per percentage-point decrease. Events: 66/106 developed T2DM
Xia Y et al., 2022 [33]	China; TCLSIH cohort; prospective cohort; adults assessed for NAFLD, diet, and incident T2DM; n = 24,602	93,873 person-years; liver ultrasonography with alcohol consumption considered	Incident T2DM during follow-up; exact criteria not stated in the abstract	NAFLD: HR 3.04 (95% CI 2.51-3.68); low vegetable intake: HR 4.08 (3.05-5.46); high vegetable intake: HR 2.38 (1.85-3.07); interaction P < 0.01. Events: 787 incident T2DM cases
Bae JC et al., 2018 [34]	Republic of Korea; Kangbuk Samsung Health Study; longitudinal health-check cohort; adults without T2DM undergoing annual comprehensive health checks; n = 7,849	Mean 4 years, with annual assessments over 5 years; annual abdominal ultrasonography	Incident diabetes during follow-up; exact criteria not stated in the abstract	Sustained versus never NAFLD: HR 1.50 (95% CI 1.13-1.98); intermittent versus never NAFLD: HR 0.99 (0.76-1.31); sustained versus intermittent NAFLD: HR 1.50 (1.20-1.89). Events: not reported in the PubMed abstract
Tokita Y et al., 2017 [35]	Japan; Hidaka Hospital health-check population; 10-year observational health-check cohort; 1,544 men and 864 women without impaired glucose tolerance; excessive alcohol consumption excluded; n = 2,408	10 years; abdominal ultrasonography; alcohol intake >20 g/day excluded	Development of diabetes during follow-up; exact criteria not stated in the abstract	RR 4.8 (95% CI 3.0-7.8) in men and 14.5 (7.0-30.1) in women. Events: 88 in total—29/232 versus 34/1,312 in men and 10/38 versus 15/826 in women
Zheng X et al., 2021 [36]	Japan; Murakami Memorial Hospital cohort; retrospective propensity score-matched cohort; 14,280 adults without diabetes; 1,671 matched participants per exposure group; n = 14,280	Observation window from 2004 to 2015; exact individual follow-up not reported in the abstract; NAFLD diagnostic method not stated in the PubMed abstract	Incident diabetes during follow-up; exact criteria not stated in the abstract	Matched-analysis HR 1.98 (95% CI 1.41-2.80); multivariable matched-cohort HR 2.33 (1.63-3.32); PS-adjusted HR 1.95 (1.39-2.75). Events: not reported in the PubMed abstract
Lee J et al., 2019 [37]	Republic of Korea; prediabetes health-check cohort; longitudinal health-check cohort; adults with prediabetes, including 2,830 with NAFLD; n = 6,240	Follow-up visits between 2008 and 2013; summary duration not stated in the abstract; NAFLD diagnostic method not stated in the PubMed abstract	Incident diabetes during follow-up; exact criteria not stated in the abstract	NAFLD: adjusted RR 1.81 (95% CI 1.47-2.21); WC-change tertiles: RR 1.64 (1.08-2.49), 1.73 (1.28-2.34), and 2.04 (1.42-2.93). Events: 505 incident diabetes cases
Sinn DH et al., 2019 [38]	Republic of Korea; regular health-screening cohort; large health-screening cohort; adults without diabetes, liver disease, or cancer at baseline; n = 51,463	Median 4.0 years; 236,446.6 person-years; fatty liver diagnosed using ultrasonography; severity additionally assessed using the NAFLD fibrosis score	Development of diabetes during follow-up; exact criteria not stated in the abstract	Compared with lean participants without NAFLD: lean NAFLD, HR 1.18 (95% CI 1.03-1.35); overweight/obese without NAFLD, HR 1.06 (0.98-1.14); overweight/obese with NAFLD, HR 1.45 (1.34-1.57); lean NAFLD with intermediate/high NFS, HR 2.73 (2.10-3.55). Events: 5,370 incident diabetes cases
Ming J et al., 2015 [39]	China; Xi’an/China National Diabetes and Metabolic Disorders Survey; population-based cohort; healthy adults with and without ultrasound-defined NAFLD; n = 508	5 years; abdominal ultrasonography	T2DM and prediabetes diagnosed using an oral glucose tolerance test	Adjusted RR for T2DM 4.462 (95% CI 1.855-10.734); adjusted RR for prediabetes 1.642 (0.965-2.793). Events: 20 incident T2DM cases and 85 incident prediabetes cases
Lago-Sampedro A et al., 2024 [40]	Spain; Di@bet.es; prospective cohort secondary analysis; adults with MAFLD and without T2DM at baseline; n = 714	7.5 years; MAFLD based on fatty liver index evidence of steatosis plus overweight/obesity or metabolic dysfunction	Incident T2DM assessed using OGTT, fasting biochemistry, HbA1c, diagnosis, or medication information	Adjusted OR 0.52 (95% CI 0.31-0.87) for high versus low adherence; OR 0.35 (0.16-0.78) among participants without weight gain. Events: 98
Cho Y et al., 2023 [41]	Republic of Korea; Kangbuk Samsung Health Study; retrospective longitudinal cohort; adults without diabetes or secondary causes of ultrasound-diagnosed steatosis; n = 246,424	Median 5.5 years; ultrasound-diagnosed hepatic steatosis; NAFLD and MAFLD criteria applied	Fasting glucose ≥126 mg/dL, HbA1c ≥6.5%, or glucose-lowering medication use	Women: HR 2.39 (95% CI 1.63-3.51) for NAFLD-only and 5.75 (5.17-6.36) for MAFLD. Men: HR 1.53 (1.25-1.88) and 2.60 (2.44-2.76), respectively. Events: 8,402
Chen C et al., 2023 [42]	China; Shanghai community cohort; longitudinal cohort; adults without diabetes at baseline; n = 2,690	Median 3.5 years; abdominal ultrasonography; severity additionally assessed using Gholam’s model	Incident diabetes determined using a 75-g OGTT	Incident NAFLD versus consistently non-NAFLD: OR 1.43 (95% CI 1.10-1.86). Remission versus sustained NAFLD: OR 0.48 (0.29-0.80). Events: 484
Lopes GW et al., 2024 [43]	Brazil; ELSA-Brasil; prospective cohort; civil servants aged 35-74 years without diabetes at baseline; n = 7,073	Median 9.43 years; ultrasound-detected steatosis; MASLD required steatosis plus at least one cardiometabolic factor	Self-reported diagnosis, diabetes medication use, fasting glucose, 2-h OGTT glucose, or HbA1c	Adjusted HR 1.78 (95% CI 1.58-2.01) for NAFLD and 1.88 (1.67-2.12) for MASLD. Events: 1,157
Bae JH et al., 2025 [44]	Republic of Korea; Korean Genome and Epidemiology Study; retrospective community-based cohort analysis; adults with normal glucose tolerance; n = 6,083	Median 12.4 years; observation period of up to 18 years; fatty liver index calculated using triglycerides, BMI, GGT, and waist circumference	Fasting glucose ≥126 mg/dL, 2-h OGTT glucose ≥200 mg/dL, or antidiabetic medication use	High versus low FLI: HR 3.42 (95% CI 2.91-4.00); after adjustment for insulin sensitivity and secretion markers: HR 1.96 (1.54-2.50). Events: 1,006
Xie J et al., 2024 [45]	China; annual health-check cohort; retrospective cohort; 6,559 male smokers, including 1,409 with NAFLD and 5,150 without NAFLD; n = 6,559	7 years; 39,259 person-years; abdominal ultrasonography using Chinese Liver Disease Association criteria	Fasting glucose ≥126 mg/dL, HbA1c ≥6.5%, or self-reported clinician-diagnosed T2DM	Among participants with NAFLD, HRs versus current smokers were 0.41, 2.39, 4.48, and 6.42 across increasing post-cessation weight-gain categories. Events: 363
Corbin KD et al., 2023 [46]	United States; D2d trial; post hoc longitudinal analysis of a multicenter randomized trial; adults with prediabetes randomized to receive vitamin D or placebo; n = 2,423	Median 2.5 years; validated noninvasive steatosis and fibrosis scores; no direct imaging or histology	Incident T2DM during the D2d prevention trial	NAFLD-LFS: HR 1.35 (95% CI 1.07-1.70). APRI: HR 2.36 (1.23-4.54). Vitamin D did not modify the associations. Events: not reported in the extracted abstract
Sakai K et al., 2024 [47]	Japan; NAGALA, Asahi University Hospital; retrospective health-check cohort; adults undergoing medical health checkups; n = 15,039	5 years; hepatic steatosis assessed using abdominal ultrasonography; NAFLD and MASLD criteria applied	Fasting glucose ≥126 mg/dL, HbA1c ≥6.5%, or specific diabetes treatment	Adjusted OR 6.00 (95% CI 4.84-7.45) for NAFLD and 6.58 (5.29-8.17) for MASLD; AUC 0.799 versus 0.807. Events: NAFLD, 230/2,785; MASLD, 234/2,682
Li N et al., 2023 [48]	China; Karamay health-check cohort; retrospective cohort; young and middle-aged adults without diabetes at baseline; n = 3,001	2 years; abdominal ultrasonography; lean defined as BMI <25 kg/m²	Incident T2DM during annual health examinations	Lean NAFLD versus lean non-NAFLD: adjusted HR 3.83 (95% CI 2.02-7.24). Events: not clearly reported in the extracted abstract
Si R et al., 2024 [49]	Japan; NAGALA; secondary longitudinal cohort analysis; normoglycemic adults; n = 14,443	Mean 6.1 years; hepatic steatosis index derived from the ALT/AST ratio, BMI, sex, and diabetes status	Incident T2DM in the NAGALA follow-up dataset	Compared with Q1, adjusted HRs were 1.09 (95% CI 0.61-1.93), 1.16 (0.68-1.98), and 3.30 (2.04-5.33) for Q2, Q3, and Q4, respectively. Events: 238
Park SH et al., 2023 [50]	Republic of Korea; Samsung health-check cohort; retrospective longitudinal cohort; adults with at least two serial health examinations; n = 21,178	Median 5.0 years; hepatic steatosis determined using abdominal ultrasonography	Incident diabetes during serial health examinations	Compared with non-FLD without metabolic dysfunction: HR 2.68 (95% CI 1.58-4.53) for NAFLD-only, 6.17 (4.96-7.68) for both definitions, and 8.30 (6.13-11.24) for MAFLD-only. Events: 1,296
Han WM et al., 2023 [51]	Thailand; HIV-NAT cohort; prospective cohort; adults with HIV without excessive alcohol use or hepatitis coinfection; n = 847	2013-2022; 2,552 person-years; NAFLD defined as CAP ≥248 dB/m; NASH with activity and fibrosis defined as FAST score ≥0.67	Fasting glucose ≥126 mg/dL on two visits, HbA1c ≥6.5%, or reported diagnosis with treatment initiation	NAFLD: adjusted HR 2.83 (95% CI 1.26-6.36). NAFLD plus NASH: adjusted HR 3.12 (1.05-9.26). Events: incidence of 10.9 per 1,000 person-years
Liang X et al., 2025 [52]	Japan; NAGALA, Murakami Memorial Hospital; secondary cohort analysis; normoglycemic adults with NAFLD; n = 2,506	Up to 11 years; NAFLD in the NAGALA dataset	Self-reported physician diagnosis, HbA1c ≥6.5%, or fasting glucose ≥7.0 mmol/L	U-shaped association with an inflection point at TyG 7.82. Below the threshold: HR 0.07 (95% CI 0.01-0.32); above the threshold: HR 1.70 (1.19-2.44) per unit. Events: 203
Xie X et al., 2023 [53]	Japan; NAGALA; secondary population-based cohort analysis; normoglycemic Japanese men with NAFLD; n = 1,221	Median 6.05 years; NAFLD in the NAGALA dataset	Fasting glucose ≥7 mmol/L, HbA1c ≥6.5%, or self-report	U-shaped association. Below a TG level of 53 mg/dL: HR 0.413 (95% CI 0.220-0.778) per 10 mg/dL. Above the threshold: HR 1.091 (1.046-1.137). Events: 39
Cho Y et al., 2023 [54]	Republic of Korea; Kangbuk Samsung Health Study; retrospective cohort; parous women without diabetes at baseline; n = 97,347	Median 3.9 years; ultrasound-diagnosed NAFLD	Incident T2DM during serial health assessments	HR 2.61 (95% CI 2.06-3.31) for prior GDM for prior GDM alone, 2.26 (1.96-2.59) for NAFLD alone, and 6.45 (5.19-8.00) for both. Events: 1,515
Hatano Y et al., 2023 [55]	United States; CARDIA; population-based prospective cohort; Black and White adults without diabetes at baseline; n = 1,995	Approximately 5 years; noncontrast CT liver attenuation ≤51 Hounsfield units after excluding other causes	Fasting glucose ≥7.0 mmol/L or diabetes medication use at follow-up	Adjusted risk difference of 4.1% (95% CI 0.3%-7.9%) in White participants and -1.9% (-5.7% to 2.0%) in Black participants. Events: 3.7% of White and 8.0% of Black participants developed T2DM
Anastasiou G et al., 2023 [56]	Greece; retrospective statin-initiation cohort; adults with dyslipidemia without baseline T2DM; n = 882	Median or typical follow-up of approximately 6 years (IQR 4-10); fatty liver index; probable NAFLD defined as FLI ≥60	New-onset T2DM during clinical follow-up	Probable NAFLD (FLI ≥60) was associated with incident T2DM (adjusted HR 3.14, 95% CI 1.50-6.59); approximately 11% developed T2DM
Petroni ML et al., 2023 [57]	Italy; clinical NAFLD lifestyle-intervention cohorts; 546 enrolled, including 363 without diabetes at baseline	Up to 60 months, with visits every 6-12 months; established NAFLD at enrollment	Diabetes newly diagnosed during scheduled clinical follow-up	Forty-eight new diabetes cases occurred. Weight reduction was inversely associated with incident diabetes (OR 0.57, 95% CI 0.41-0.79); web- and group-based programs had similar diabetes incidence
Faria LC et al., 2023 [58]	Brazil; ELSA-Brasil; prospective adult cohort without diabetes at baseline	Prospective follow-up; liver steatosis assessed at baseline using a validated steatosis assessment in ELSA-Brasil	Incident diabetes assessed using ELSA-Brasil follow-up glycemic and clinical criteria	Baseline liver steatosis was associated with a higher subsequent incidence of diabetes after multivariable adjustment
Sinn DH et al., 2023 [59]	Republic of Korea; Samsung longitudinal health-screening cohort; adults with NAFLD at the initial examination; n = 11,260	NAFLD reassessed after 1-2 years; median diabetes follow-up of 4 years; abdominal ultrasonography	Incident diabetes during longitudinal health-screening follow-up	NAFLD regression versus persistence was associated with a lower risk of incident diabetes (adjusted HR 0.81, 95% CI 0.72-0.92); 1,768 cases occurred
Shih CI et al., 2024 [60]	Taiwan; large health-check cohort with a cross-sectional baseline and longitudinal follow-up; adults free of diabetes for the incidence analysis	Mean follow-up of approximately 6.75 years for diabetes; fatty liver severity assessed using ultrasonography	New-onset diabetes during follow-up	Mild and moderate/severe fatty liver were associated with graded diabetes risk (adjusted HR 3.22, 95% CI 2.56-4.07, and 5.88, 4.44-7.81, respectively)
Park KY et al., 2024 [61]	Republic of Korea; Korean National Health Insurance Service; young adults aged 20-39 years with prediabetes; n = 446,813	Median 8.3 years; two health examinations; NAFLD defined as FLI ≥60, with changes in status classified	Incident T2DM identified using national claims and health-screening data	Persistent NAFLD showed the highest diabetes risk; adjusted HR 5.38 versus persistent non-NAFLD; 26,464 incident T2DM cases
Ha J et al., 2024 [62]	Republic of Korea; Korean National Health Insurance Service health-screening cohort; adults aged 20-39 years; n = 6,232,656	Median 9.5 years; FLI and metabolic criteria used to classify MAFLD/NAFLD groups	Incident diabetes identified using claims data	MAFLD was associated with incident diabetes (adjusted HR 6.148, 95% CI 6.084-6.212); 182,291 incident cases
Wu C et al., 2024 [63]	Japan; NAGALA/Murakami Memorial Hospital health-check cohort; adults without baseline diabetes; n = 15,464	93,350 person-years; median 5.9 years; ZJU index used as a surrogate NAFLD-related metabolic index	Incident diabetes during NAGALA follow-up	The highest versus lowest ZJU-index quartile remained associated with diabetes after adjustment (HR 2.519, 95% CI 1.297-4.891); 373 cases
Wang Y et al., 2025 [64]	Japan; NAGALA health-check cohort; adults without baseline T2DM; n = 15,464	Approximately 8-year analytical horizon; ultrasound-detected fatty liver and clinical/metabolic predictors	Incident T2DM during longitudinal follow-up	Fatty liver was retained among the predictors of incident T2DM in a multivariable prediction model with high reported discrimination
Chen Y et al., 2025 [65]	Japan; secondary retrospective NAFLD cohort from an established health-check dataset; n = 2,370	Longitudinal follow-up; NAFLD present at baseline; AIP evaluated as a within-NAFLD risk marker	Incident T2DM during follow-up	The highest versus lowest AIP tertile was associated with incident T2DM (adjusted OR 1.99, 95% CI 1.29-3.08); continuous AIP: OR 2.76 (1.54-4.93)
Yang L et al., 2025 [66]	Japan; longitudinal NAFLD subgroup from a large health-check cohort; n = 2,741 with NAFLD	Longitudinal health-check follow-up; ultrasound-defined NAFLD; remnant cholesterol evaluated	New-onset T2DM during follow-up	Higher remnant cholesterol was associated with incident T2DM in NAFLD; 233 incident cases were reported in the NAFLD subgroup
Sun T, Liu J, 2025 [67]	China; single-center retrospective NAFLD cohort; n = 457	3 years; NAFLD cohort, with TyG and TyG/HDL-C evaluated as metabolic risk markers	Diabetes during longitudinal follow-up	TyG and TyG/HDL-C were associated with a higher risk of diabetes (adjusted OR 1.96 and 1.90, respectively); nonlinear relationships were reported
Cao C et al., 2025 [68]	Japan; retrospective cohort of adults with MASLD; n = 2,507	Mean 6.0 years; baseline MASLD cohort; TyG index evaluated	Incident T2DM during follow-up	Higher TyG was associated with T2DM (adjusted HR 1.48, 95% CI 1.05-2.09); a U-shaped relationship was reported; 204 cases
Hou S et al., 2025 [69]	Japan; longitudinal NAFLD cohort derived from health-check data; n = 2,503	Longitudinal follow-up; NAFLD at baseline; cardiac metabolic index evaluated	New-onset diabetes during follow-up	The highest versus lowest CMI quartile was associated with higher odds of new-onset diabetes (OR 3.239, 95% CI 1.993-5.264); 204 cases
Choque Vargas C et al., 2025 [70]	Clinical biopsy-confirmed MASLD cohort; n = 220	Approximately 10-year historical follow-up; basal fibrosis categorized using liver biopsy	Incident diabetes during long-term clinical follow-up	Advanced basal fibrosis was associated with a greater incidence of diabetes; reported adjusted HR of approximately 3.04 for F3-F4 versus F0-F2
Shih CI et al., 2025 [71]	Taiwan; health-check cohort; 32,569 cross-sectional participants and 27,109 adults free of hypertension or diabetes followed longitudinally	Longitudinal follow-up using health examinations; MASLD versus non-SLD classified using steatosis and cardiometabolic risk factors	Incident diabetes during follow-up	Diabetes incidence was substantially higher in MASLD than in non-SLD (reported rates of approximately 6.3 versus 0.6 per 1,000 person-years), with risk varying according to cardiometabolic-risk burden
Hou C et al., 2025 [72]	United Kingdom; UK Biobank transition analysis; n = 377,757 initially free of NAFLD or major CKM outcomes; 4,949 developed NAFLD	Median 13.6 years; incident NAFLD followed by transitions to cardiovascular-kidney-metabolic outcomes	First-onset T2DM after the development of NAFLD among CKM transitions	A higher insulin-resistance burden accelerated progression from NAFLD to T2DM; reported HR 1.41 (95% CI 1.11-1.78) for the relevant transition
Zhou XD et al., 2026 [73]	Three Asian MASLD cohorts: Wenzhou Real-World, Hong Kong CDARS, and SingHealth Diabetes Registry; large-scale validation study	Longitudinal cohort follow-up; MASLD clusters defined using age, BMI, HbA1c, ALT, LDL-C, and triglycerides	New-onset T2DM evaluated in cohorts with participants at risk at baseline	The cardiometabolic MASLD cluster showed the highest risk of new-onset T2DM (WRW: HR 3.418; Hong Kong CDARS: HR 2.761 versus the control cluster)
Chung GE et al., 2026 [74]	Republic of Korea; nationwide Korean National Health Insurance Service cohort; adults aged 20-39 years; n = 6,250,145	Median approximately 10.6 years; FLI-based steatotic liver disease subtypes classified using cardiometabolic and alcohol criteria	Incident T2DM identified using national longitudinal records	MASLD and other steatotic liver disease subtypes were associated with a higher incidence of T2DM; 72,028 incident cases were reported
Huo Z et al., 2026 [75]	China; Kailuan prospective cohort; 19,378 incident MASLD cases matched with 19,483 MASLD-free controls after exclusions	Median 11.8 years; MASLD onset age identified using serial health examinations	Incident T2DM during follow-up through December 31, 2020	Earlier MASLD onset conferred a greater risk of T2DM: HR 6.18 for onset before age 40 years, with elevated risks persisting across older onset groups; 2,301 cases
Chen N et al., 2026 [76]	Japan; NAGALA/Murakami Memorial Hospital; secondary longitudinal cohort analysis; adults with NAFLD at baseline; n = 2,370	12-year prospective cohort analysis; metabolic composite indices, including TyG-WHtR, assessed	Incident T2DM during prospective follow-up	TyG-WHtR showed the strongest predictive performance among the evaluated metabolic composite indices and was independently associated with incident T2DM
Wei C et al., 2026 [77]	Japan; NAGALA/Murakami Memorial Hospital; secondary longitudinal health-check analysis; adults with NAFLD and without diabetes at baseline; n = 2,741	Median 5.21 years; NAFLD at baseline; triglyceride-glycated hemoglobin index evaluated	Incident diabetes during longitudinal follow-up	Higher TyH-i was associated with an increased risk of diabetes and improved risk discrimination relative to comparator metabolic indices; 223 incident cases

The completed PRISMA 2020 reporting checklist is provided in Appendix 1. Additional details of the documented database search strategies are provided in Appendix 2.

Diagnostic and Outcome Ascertainment

USG remained the most common direct liver assessment method. Other reports used computed tomography, controlled attenuation parameter, biopsy or histology, quantitative liver fat measurement, FLI, hepatic steatosis index, and related noninvasive or surrogate scores [17-77]. The corrected update added several index-based and within-NAFLD risk-marker studies, including FLI, the ZJU index, Atherogenic index of plasma (AIP), TyG-based indices, cardiometabolic index (CMI), remnant cholesterol, and data-driven MASLD clusters [56,63-69,73,76,77]. These indices incorporate metabolic variables linked to diabetes and were therefore interpreted primarily as risk-stratification evidence rather than as proof of an independent hepatic effect.

Incident T2DM ascertainment varied across fasting glucose, HbA1c, oral glucose tolerance testing, medication use, physician diagnosis, claims data, self-report, registry linkage, and combinations of these criteria. The largest national cohorts used claims or health-screening data [20,61,62,74], whereas clinical and health-check cohorts often used laboratory definitions. Prediction-focused reports treated T2DM as a modeled endpoint and were synthesized separately from direct etiological comparisons.

Association Estimates and Structured Synthesis

Across adequately reported studies, NAFLD, MAFLD, MASLD, or related steatotic liver disease definitions were generally associated with higher estimates of incident T2DM, although the magnitude and precision varied. The corrected update reinforced this direction across independent settings, including FLI-defined probable NAFLD in statin initiators [56], liver steatosis in ELSA-Brasil [58], fatty liver severity in Taiwan [60], and very large Korean national cohorts [61,62,74]. Ha J et al. reported an adjusted HR of 6.148 for MAFLD in young adults [62], while Huo Z et al. found the greatest T2DM risk among participants with MASLD onset before 40 years of age (HR 6.18), with attenuation across older onset groups [75]. These large effects should be interpreted in light of index-based exposure definitions, metabolic overlap, and cohort non-independence.

Dynamic-status and severity studies added clinically relevant context. Sinn DH et al. reported a lower risk of diabetes after NAFLD regression than with persistent NAFLD (HR 0.81) [59], consistent with earlier dynamic-status reports [19,34,42]. Shih CI et al. observed a graded association across mild and moderate/severe fatty liver [60], and Choque Vargas C et al. reported a higher incidence of diabetes with advanced basal fibrosis in a biopsy-confirmed MASLD cohort [70]. Several newer within-NAFLD studies also identified risk gradients for AIP, remnant cholesterol, TyG-related indices, CMI, and cluster-defined metabolic phenotypes [65-73,76,77], but many used overlapping Japanese health-check data and therefore did not constitute independent replications.

A structured effect-estimate matrix is provided in Appendix 6. Adjusted HRs, RRs, and ORs are displayed separately in Appendices 9-11, without pooled estimates. The post-extraction feasibility assessment did not identify a clinically defensible group of independent studies for quantitative pooling because exposure definitions, populations, comparators, outcome methods, adjustment sets, and effect measures remained heterogeneous, and several datasets overlapped.

Risk of Bias and Confidence in the Evidence

Study-level NOS judgments for all 61 included reports are presented in Appendix 4 and Appendix 9. Most reports were judged to be at low or moderate risk, with lower ratings generally reflecting selected clinical populations, surrogate or index-based exposure definitions, limited comparability, prediction-focused analyses, or incomplete outcome information. The 22 newly identified reports were appraised using the same review-specific NOS thresholds as the previously included evidence. Because several reports were secondary analyses of NAGALA, Korean national health-screening, Samsung, ELSA-Brasil, or Taiwanese datasets, apparent consistency across publications was not treated as independent replication.

Discussion

Summary of Principal Findings

This updated systematic review included 61 longitudinal reports. Across adequately reported studies, the direction of the association between steatotic liver disease and incident T2DM was generally positive, but the magnitude varied substantially across exposure definitions, populations, follow-up structures, outcome methods, and adjustment strategies. The corrected supplementary search added recent longitudinal evidence through July 2026, including large national cohorts, dynamic MASLD analyses, and multiple within-disease metabolic risk-marker studies [56-77]. The expanded evidence strengthened the overall interpretation of steatotic liver disease as a metabolic risk marker while also increasing the importance of accounting for overlapping cohorts and metabolically derived indices.

The findings support three cautious interpretations. First, steatotic liver disease is a clinically relevant marker of future metabolic deterioration, but the available observational evidence does not establish a uniform independent or causal effect. Second, a higher occurrence of diabetes was reported across several BMI-defined groups, including non-overweight, non-obese, and lean populations, although these categories were defined differently and should not be treated as a single phenotype. Third, persistent or newly developed disease, greater liver fat burden, and advanced fibrosis were associated with higher estimates of future diabetes occurrence in some cohorts, whereas remission or a reduction in liver fat was associated with lower estimates. These patterns support a risk-marker interpretation rather than individualized prediction or confirmed reversibility of diabetes risk.

NAFLD as a Metabolic Risk Marker

The original evidence showed a higher incidence of diabetes among adults with fatty liver than among comparison groups [17,21,30]. Adams LA et al. [17] reported attenuation after adjustment for waist circumference, hypertension, and insulin resistance. This attenuation suggests that part of the association reflects a broader insulin-resistant metabolic profile rather than an isolated liver-specific effect. However, adiposity, waist circumference, and insulin resistance may act as confounders, mediators, or both. Extensive adjustment may therefore reduce confounding while also attenuating the pathways through which steatotic liver disease is associated with T2DM.

Large cohorts provided additional evidence but require cautious clinical interpretation. Chung GE et al. [20] reported an almost fivefold adjusted association in young adults, while Kim Y et al. [24] found that NAFLD improved diabetes risk prediction beyond conventional factors in their cohort. These large relative estimates identify potentially higher-risk groups, but their clinical application requires corresponding absolute risk information and validation in independent populations. The evidence therefore supports considering liver status as one component of a broader metabolic assessment rather than as a stand-alone prediction tool.

NAFLD Across BMI-Defined Groups

Findings outside conventional obesity categories were clinically important but heterogeneous. Fukuda T et al. [22] evaluated non-overweight adults, Dai W et al. [25] studied a non-obese cohort, Fan JG et al. [21] reported findings in non-obese participants with fatty liver, and Li N et al. [48] examined lean NAFLD defined by a BMI below 25 kg/m². These study-defined groups differed in BMI thresholds, ethnicity, baseline metabolic health, liver disease ascertainment, and adjustment methods.

Collectively, the findings suggest that hepatic steatosis may identify vulnerability to diabetes that is not fully captured by BMI alone. They do not, however, establish non-overweight, non-obese, and lean NAFLD as a single uniform risk category. Central adiposity, insulin resistance, dyslipidemia, genetic susceptibility, and other metabolic abnormalities may remain present despite a BMI below conventional obesity thresholds.

Dynamic NAFLD Status and Attenuation of Diabetes Risk

Most included reports assessed liver status at baseline only, but studies with repeated assessments provided additional context. Cho HJ et al. [19], Bae JC et al. [34], and Chen C et al. [42] reported higher estimates of diabetes occurrence with persistent or newly developed NAFLD and lower estimates after improvement or resolution. The corrected update added Sinn DH et al. [59], in whom regression of ultrasound-defined NAFLD was associated with a lower risk of incident diabetes than persistent NAFLD, and Park KY et al. [61], in whom persistent FLI-defined NAFLD among young adults with prediabetes carried the highest subsequent T2DM risk. Huo Z et al. further showed that earlier MASLD onset was associated with progressively greater T2DM risk [75].

These findings support an attenuation of diabetes risk associated with NAFLD improvement rather than confirmed reversibility. Remission or a reduction in liver fat may occur alongside weight loss, improved insulin sensitivity, dietary changes, increased physical activity, or other metabolic improvements. The observational designs therefore cannot establish that changing liver fat alone caused the reduction in diabetes occurrence.

Histologic Steatosis, Fibrosis Stage, and Liver Fat Burden

Biopsy-based reports evaluated related but noninterchangeable dimensions of liver disease. Kogiso T et al. [23] described de novo diabetes in a selected biopsy-confirmed NAFLD cohort. Nasr P et al. [32] found that steatosis grade and quantified liver fat were associated with subsequent T2DM, while Sanyal AJ et al. [29] reported a higher incidence of T2DM in participants with F4 fibrosis than in those with F0-F2 fibrosis. Updated studies also reported associations using noninvasive fibrosis or steatosis measures, including the NAFLD liver fat score, APRI, controlled attenuation parameter, and combined NASH-fibrosis criteria [46,51].

These findings suggest that future diabetes occurrence may vary according to the type and severity of the liver abnormality, but steatosis, quantified liver fat, and fibrosis should not be interpreted as representing the same metabolic pathway. Direct comparisons are limited by differences in assessment methods and by referral and spectrum bias in biopsy-based cohorts, which often include patients selected because of greater clinical concern or more advanced disease.

Demographic, Reproductive, and Lifestyle-Related Differences

Several reports described differences across demographic or clinical subgroups. Chung GE et al. [20] studied young adults, Kim Y et al. [24] reported different estimates according to sex and menopausal status, Cho Y et al. [54] found a higher joint risk among women with both NAFLD and prior gestational diabetes, and Hatano Y et al. [55] reported different estimates among Black and White CARDIA participants. These findings may identify clinically relevant heterogeneity, but subgroup effects were not consistently replicated across independent populations.

Lifestyle and behavioral factors were associated with differences in diabetes occurrence among participants with NAFLD. Poor sleep and insomnia were examined by Zhang H et al. [27], current smoking by Liu C et al. [31], Mediterranean diet adherence by Lago-Sampedro A et al. [40], and smoking cessation with post-cessation weight change by Xie J et al. [45]. Because these exposures and interactions were assessed inconsistently and often within single cohorts, they should be interpreted as hypothesis-generating rather than as established universal effect modifiers. Evidence relating to genetic susceptibility was also limited; Kogiso T et al. [23] reported an association involving the KCNQ1 genotype within a selected clinical cohort.

Diagnostic Definitions and Implications for Interpretation

The included reports did not define a single directly comparable liver exposure. Conventional USG identifies steatosis but may miss milder disease. Computed tomography and controlled attenuation parameter assess liver fat using different measurement principles, while biopsy provides detailed information on steatosis, inflammation, and fibrosis but is usually applied in clinically selected populations. Elevated alanine aminotransferase after the exclusion of competing causes was used as a surrogate definition in one cohort [17]; it is nonspecific, and normal aminotransferase levels do not exclude clinically important steatotic liver disease or advanced fibrosis.

Index-based definitions create a distinct interpretive problem. The FLI, hepatic steatosis index, ZJU index, TyG-based indices, AIP, CMI, and related scores incorporate metabolic variables that are themselves associated with diabetes [44,46,49,56,63-69,76,77]. Strong associations or discriminatory performance observed with these scores may therefore reflect shared metabolic information rather than liver status alone. The same caution applies to prediction models and cluster-based analyses [18,64,73]. These findings are clinically useful for risk stratification but should not be interpreted as demonstrating a stand-alone causal contribution of steatosis.

Changes in nomenclature also influence eligibility and interpretation. Updated reports compared or applied NAFLD, MAFLD, MASLD, and broader steatotic liver disease subtypes [41,43,47,50,62,68,71,73-75]. These categories overlap substantially but are not interchangeable. The review therefore retained the exposure definition used by each original report and interpreted differences in case definitions as a source of clinical heterogeneity rather than treating all labels as identical.

Clinical and Public Health Implications

The evidence supports steatotic liver disease as a marker that may prompt a broader assessment of glycemic and cardiometabolic risk. This is particularly relevant when liver disease is persistent, liver fat or fibrosis burden is greater, or additional risk factors such as prior gestational diabetes, smoking, weight gain, or adverse metabolic profiles coexist. However, the evidence does not support using NAFLD, MAFLD, or MASLD alone as a deterministic prediction tool or as a basis for automatic treatment escalation.

Clinical interpretation should emphasize comprehensive risk assessment rather than isolated liver classification. Repeated evaluation of glycemia, adiposity, blood pressure, lipids, and modifiable lifestyle factors may be appropriate as part of usual clinical care. Absolute risk estimates, external validation, and intervention studies are still needed before the observed relative associations can be translated into specific diabetes-screening intervals or prevention pathways.

Methodological Limitations of the Included Evidence

Several limitations affect the interpretation of the included evidence. Liver disease ascertainment varied across USG, computed tomography, liver indices, alanine aminotransferase elevation, controlled attenuation parameter, biopsy, and quantitative liver fat measures. This heterogeneity can change who is classified as exposed and can influence the apparent magnitude of the association. Methods for excluding diabetes at baseline also differed: some cohorts used fasting glucose alone, whereas others used HbA1c, oral glucose tolerance testing, medication records, claims data, or combinations of these approaches. Incomplete detection of subclinical dysglycemia at baseline may therefore have contributed to reverse causation or early outcome misclassification.

Outcome ascertainment and follow-up also varied. Greater contact with healthcare services among participants with known liver disease may produce surveillance bias. Loss to follow-up and complete-case selection may distort estimates when attrition is related to metabolic risk, and competing events such as death may reduce the opportunity to observe incident diabetes in older or clinically selected cohorts. These issues were not handled uniformly across reports.

Adjustment strategies require particular caution. Adiposity, waist circumference, insulin resistance, dyslipidemia, and related variables may act as confounders, mediators, components of index-based exposure definitions, or combinations of these roles. Minimally adjusted estimates may retain confounding, whereas extensively adjusted models may remove part of the pathway through which steatotic liver disease relates to diabetes. Attenuation after adjustment should therefore not be interpreted simply as proof that the association is absent or as a technical difference between models.

Many reports came from health-check, occupational, database, or specialist clinical populations, limiting generalizability. Biopsy cohorts may overrepresent more severe disease, while health-check cohorts may underrepresent people with limited healthcare access. Several updated reports arose from NAGALA or the Kangbuk Samsung Health Study and were treated as related analyses rather than independent replications. Subgroup findings, prediction thresholds, and lifestyle interactions were seldom reproduced across independent cohorts.

Limitations of This Review

This review also has limitations. The synthesis was narrative and did not generate a pooled effect estimate or a formal GRADE assessment. The protocol was not prospectively registered. The original search log did not preserve database-specific yields or the exact execution date, and the corrected supplementary search was limited to PubMed/MEDLINE and Scopus. The repeated supplementary query retrieved 35 eligible reports, while three eligible reports from the same publication period that had been included previously were not re-retrieved and were retained after their eligibility was reconfirmed; this illustrates the sensitivity limitations of pragmatic search wording and index terminology. The review also includes multiple secondary analyses from overlapping cohorts, particularly NAGALA and Korean health-screening datasets, so the publication count should not be equated with the number of independent populations.

The review included direct etiological comparisons, within-NAFLD modifier analyses, dynamic-status studies, and prediction-focused reports. These designs addressed related but different questions and were therefore synthesized in separate analytical themes rather than interpreted as equivalent evidence. Overlapping cohort reports were also not treated as independent replications. Differences in populations, nomenclature, exposure definitions, baseline diabetes screening, outcome ascertainment, and confounder adjustment limit direct comparisons and causal interpretation.

Quantitative pooling was reconsidered after structured extraction. Even within groups defined by USG, FLI, lean status, or biopsy, studies differed in comparator definitions, baseline diabetes screening, follow-up, outcome ascertainment, adjustment sets, and estimands; some also shared participants. The absence of a pooled estimate therefore reflects a lack of clinical comparability rather than a numerical heterogeneity threshold. The supplementary effect matrix and unpooled forest-style plots make the reported adjusted estimates and confidence intervals visible without implying that HRs, RRs, ORs, incidence proportions, prediction metrics, and interaction findings are equivalent.

Confidence in the principal conclusions was evaluated qualitatively according to risk of bias, consistency, directness, precision, and possible publication bias. Confidence was greatest for the general direction of the association in adequately reported longitudinal cohorts and was lower for the exact magnitude, subgroup effects, dynamic remission, and prediction performance. Publication bias could not be tested statistically; preferential publication or selective reporting of positive subgroup findings, small studies, and prediction models remains possible.

## Conclusions

This review found that NAFLD and related steatotic liver disease definitions were generally associated with a higher incidence of T2DM across diverse longitudinal settings. Persistent or newly developed steatosis, greater liver fat or fibrosis burden, and several adverse metabolic profiles were associated with higher estimates of future diabetes occurrence, whereas regression or reduction of steatosis was associated with lower estimates in some cohorts. Because definitions, adjustment strategies, and datasets varied substantially, these findings support steatotic liver disease as a clinically relevant risk marker rather than as evidence of causality or a stand-alone individualized prediction tool. Future work should prioritize standardized terminology, repeated liver assessment, transparent separation of confounders from metabolic mediators, external validation, and independent cohorts.

## Figures and Tables

**Figure 2:**
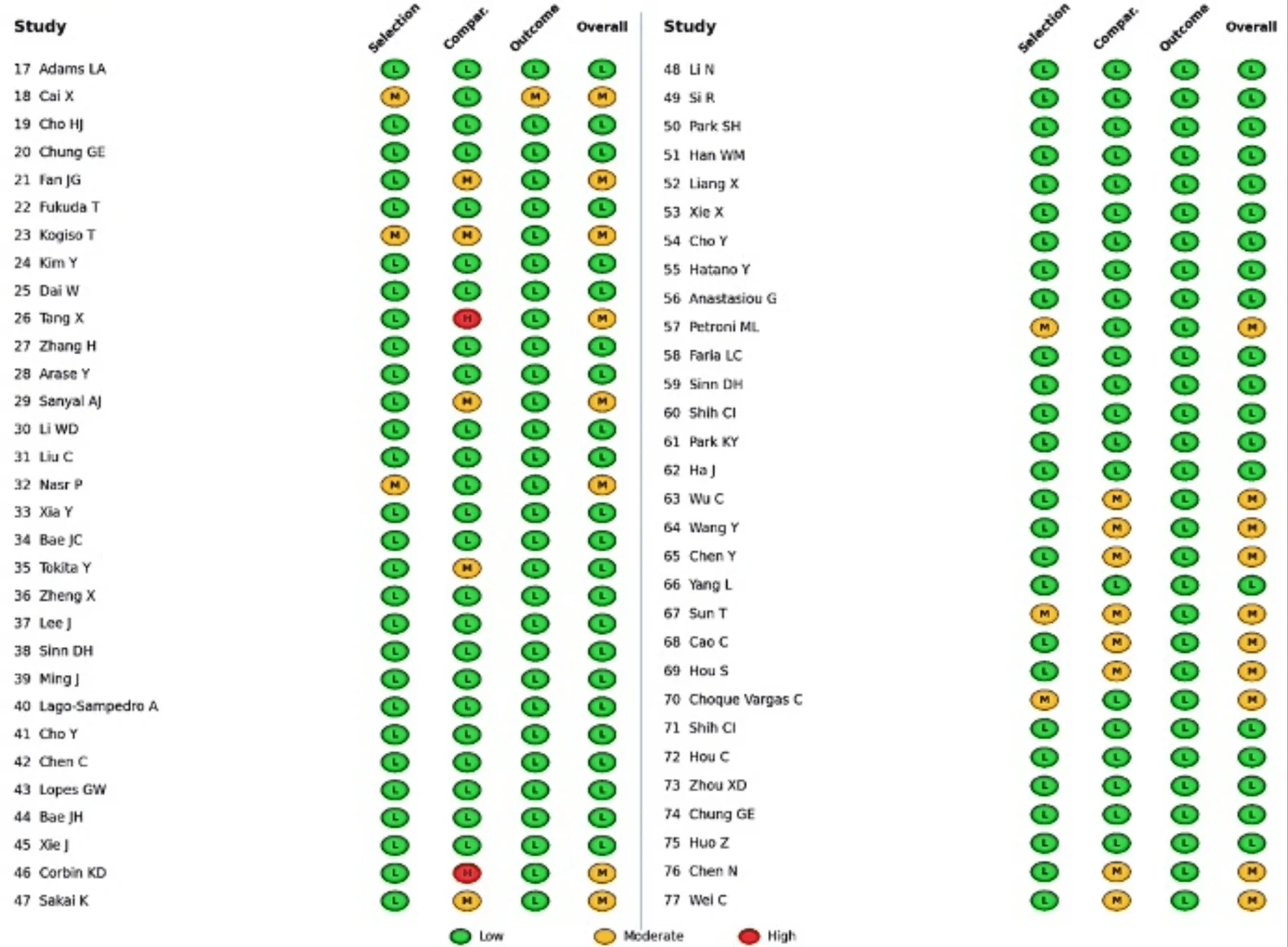
Risk-of-bias traffic-light plot for the 61 included reports. Risk of bias was assessed using the Newcastle-Ottawa Scale across selection, comparability, and outcome domains, with the overall judgment derived from the total NOS score. Low risk corresponds to 7-9 stars, moderate risk to 5-6 stars, and high risk to fewer than 5 stars. L: low risk; M: moderate risk; H: high risk; NOS: Newcastle-Ottawa Scale.

**Figure 3:**
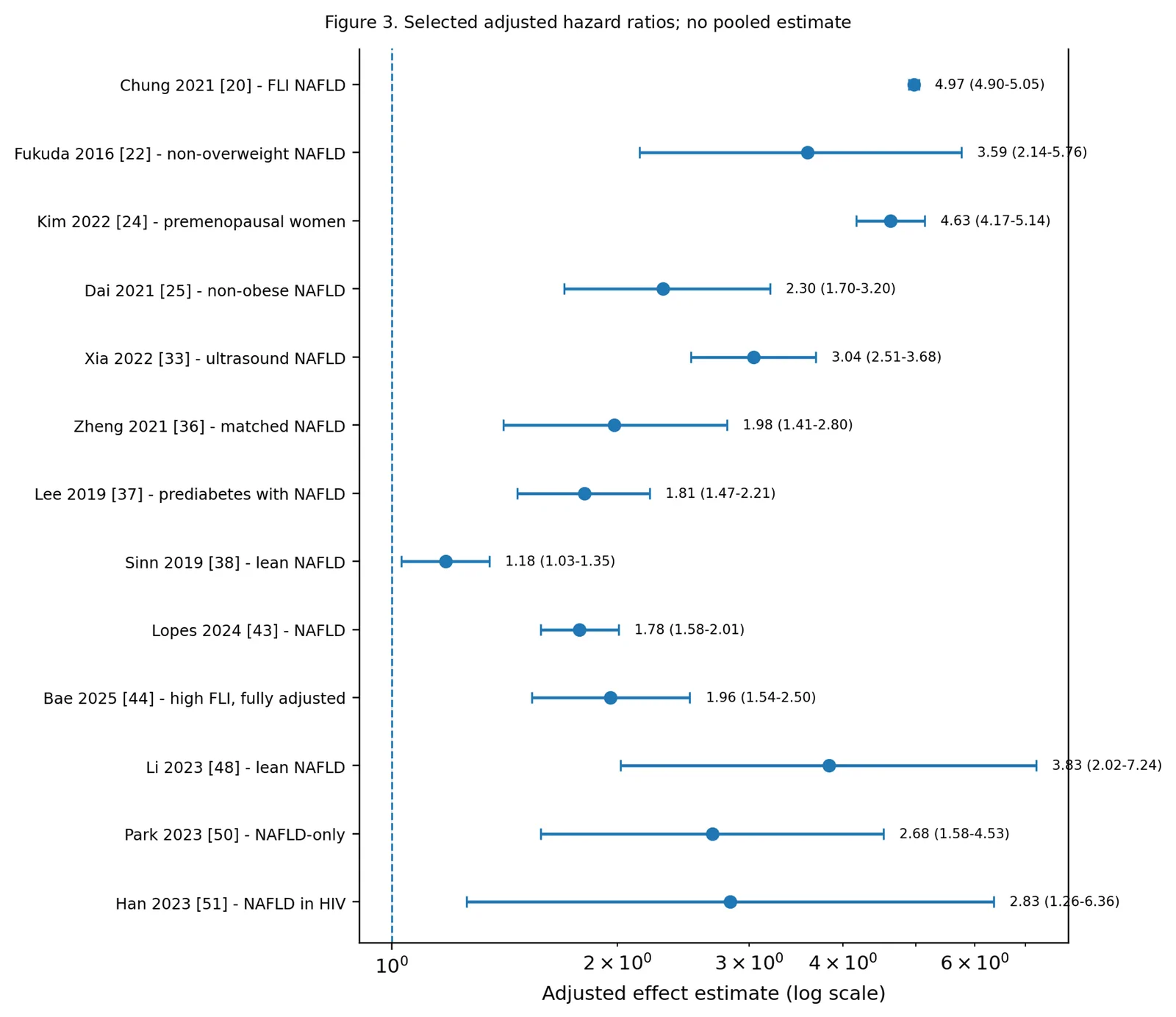
Selected adjusted HRs with 95% CIs. No pooled estimate was calculated. The figure presents selected adjusted HRs and their corresponding 95% CIs. Estimates are displayed without a pooled summary estimate because of heterogeneity in study populations, steatotic liver disease definitions, comparator groups, follow-up periods, covariate adjustment, and the use of overlapping cohort data. The vertical reference line at HR = 1 indicates no association. HR: Hazard ratio.

**Figure 4:**
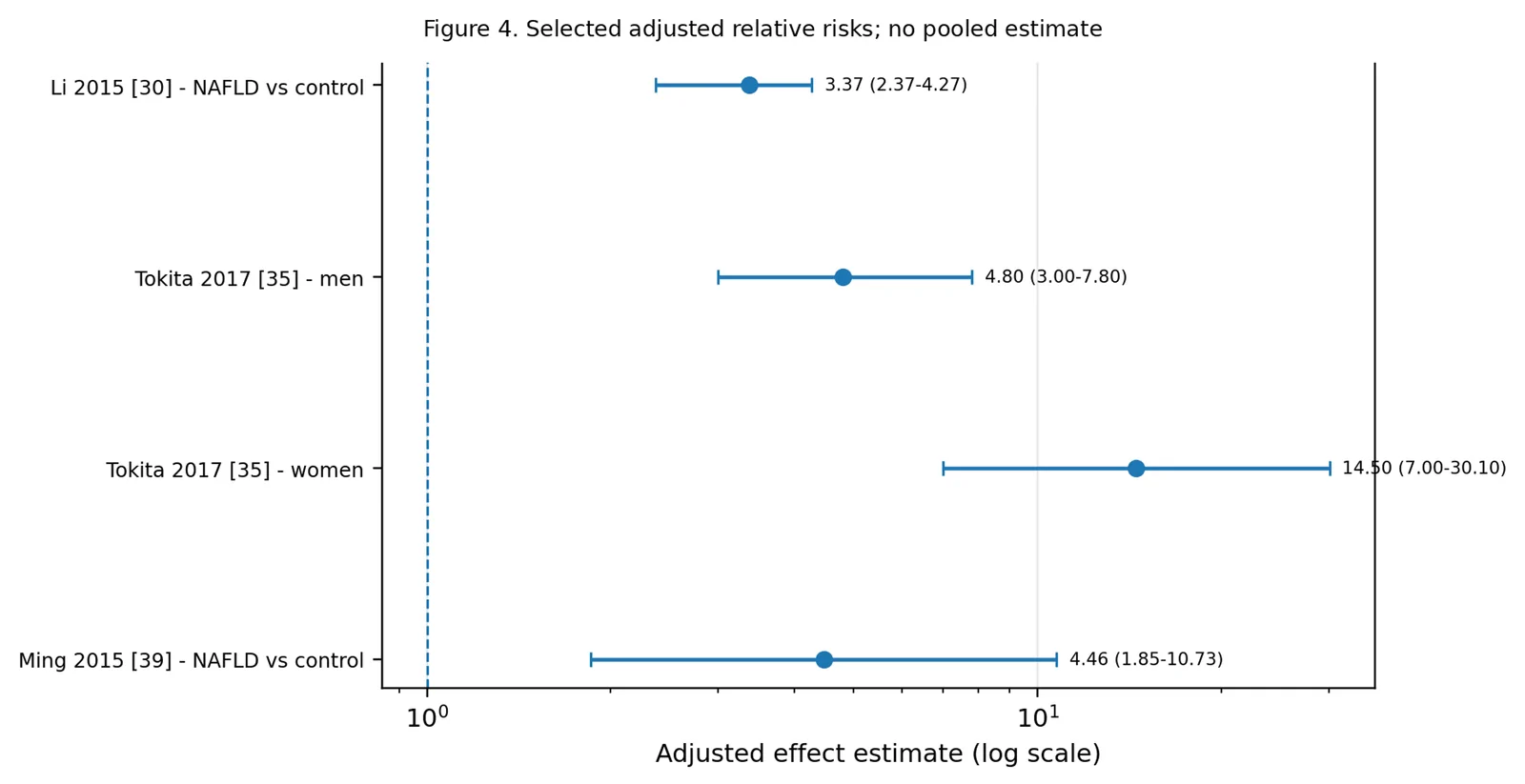
Selected adjusted relative risks for incident type 2 diabetes mellitus. The figure presents selected adjusted relative risks and their corresponding 95% CIs. Estimates are displayed without a pooled summary estimate because the studies differed in exposure definitions, populations, outcome ascertainment, follow-up durations, adjustment models, and cohort sources. The vertical reference line at RR = 1 indicates no association. RR: Relative risk.

**Figure 5:**
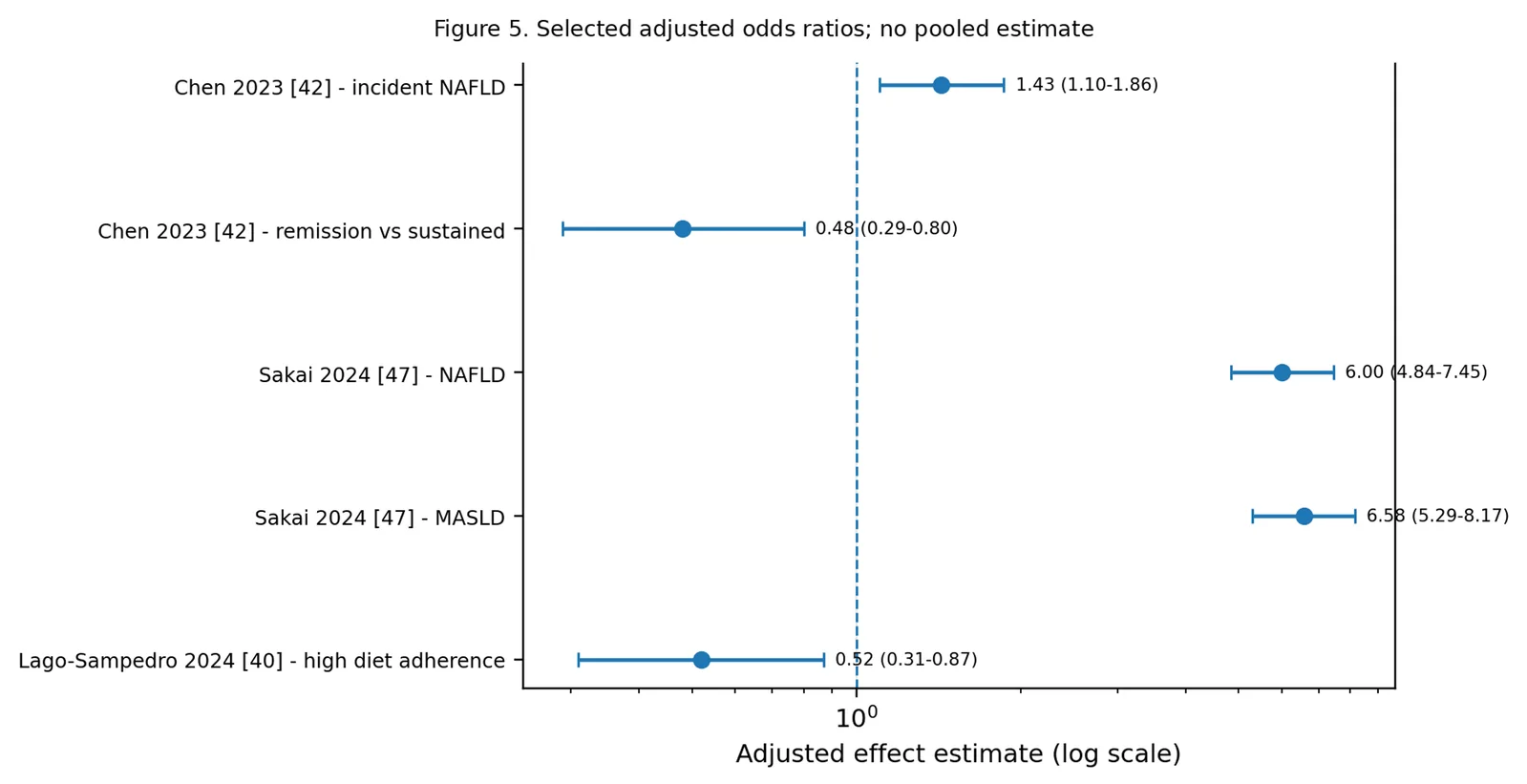
Selected adjusted odds ratios for incident type 2 diabetes mellitus. The figure presents selected adjusted ORs and their corresponding 95% CIs. Estimates are displayed without a pooled summary estimate because of clinical and methodological heterogeneity and differences in exposure comparisons and analytical models. The vertical reference line at OR = 1 indicates no association. OR: Odds ratio; T2DM: Type 2 diabetes mellitus.

## Appendices

Appendix 1

**Table 4:** PRISMA 2020 reporting checklist summary. PRISMA 2020 reporting checklist summary. This table summarizes the applicable PRISMA 2020 reporting items and identifies where each item is addressed in the manuscript and accompanying supplementary materials. PRISMA: Preferred Reporting Items for Systematic Reviews and Meta-Analyses.

PRISMA section	Item number	Checklist item	Location in manuscript	Reporting status
Title	1	Identify the report as a systematic review.	Title page	Reported
Abstract	2	Provide a structured or journal-appropriate summary of the review.	Abstract	Reported
Introduction	3	Describe the rationale for the review in the context of existing knowledge.	Introduction	Reported
Introduction	4	Provide an explicit statement of the objective or review question.	Introduction	Reported
Methods	5	Specify the eligibility criteria for the review.	Methods, Eligibility criteria; Table 2	Reported
Methods	6	Specify all information sources searched or consulted.	Methods, Information sources and search strategy	Reported
Methods	7	Present the full search strategies for all databases and other sources.	Methods, Information sources and search strategy; Table 1	Reported
Methods	8	Specify the methods used to determine whether studies met the eligibility criteria.	Methods, Study selection	Reported
Methods	9	Specify the methods used to collect data from the included reports.	Methods, Data extraction and management of incomplete information	Reported
Methods	10	List and define all outcomes and other variables for which data were sought.	Methods, Data extraction and management of incomplete information; Outcomes and analytical themes	Reported
Methods	11	Specify the methods used to assess risk of bias in the included studies.	Methods, Risk-of-bias assessment	Reported
Methods	12	Specify the effect measures sought for each outcome.	Methods, Data extraction and management of incomplete information; Data synthesis and assessment of quantitative pooling	Reported
Methods	13	Describe the methods used to synthesize the study results.	Methods, Data synthesis and assessment of quantitative pooling	Reported
Methods	14	Describe the methods used to assess reporting bias and missing results.	Methods, Certainty of evidence, reporting bias, and missing results	Reported narratively
Methods	15	Describe the methods used to assess certainty or confidence in the body of evidence.	Methods, Certainty of evidence, reporting bias, and missing results; Table 8	Reported using a structured qualitative assessment
Results	16	Describe the results of the study identification and selection process.	Results, Study selection; Figure 1; Supplementary Table 10	Reported
Results	17	Cite each included study and present its characteristics.	Results, Study characteristics; Table 3	Reported
Results	18	Present the risk-of-bias assessments for the included studies.	Results, Risk of bias and confidence in the evidence; Table 7; Figure 2	Reported
Results	19	Present the results for each outcome in each included study.	Results, Association estimates and structured synthesis; Tables 3 and 9; Figures 3-5	Reported
Results	20	Present the results of each synthesis conducted.	Results, Association estimates and structured synthesis; Table 9; Figures 3-5	Reported narratively without pooled estimates
Results	21	Present assessments of reporting bias or missing results, where applicable.	Methods, Certainty of evidence, reporting bias, and missing results; Discussion, Limitations of this review	Addressed narratively; no formal assessment was performed
Results	22	Present assessments of certainty or confidence in the body of evidence.	Results, Risk of bias and confidence in the evidence; Table 8	Reported using a structured qualitative assessment
Discussion	23	Provide an interpretation of the results in the context of other evidence, including limitations and implications.	Discussion	Reported
Other information	24	Provide registration and protocol information and describe any protocol amendments.	Methods, Review design	Partially reported: absence of prospective registration and an amendment log is stated, but protocol-access information should be added
Other information	25	Describe sources of financial or nonfinancial support.	Declarations, Funding	Reported
Other information	26	Declare competing interests of the review authors.	Declarations, Conflicts of interest	Reported
Other information	27	Report which data, code, and other materials are publicly available and where they can be accessed.	Declarations, Data availability	Reported

Appendix 2 

**Table 5:** Documented PubMed/MEDLINE and Scopus search strategies. The table presents the documented original-search record and the corrected supplementary PubMed/MEDLINE and Scopus search details, including the repeated-search record counts. The original database-specific yields and exact original execution date were not retained. MAFLD: Metabolic dysfunction-associated fatty liver disease; MASLD: Metabolic dysfunction-associated steatotic liver disease; MEDLINE: Medical Literature Analysis and Retrieval System Online; NAFLD: Non-alcoholic fatty liver disease; T2DM: Type 2 diabetes mellitus.

Database	Original coverage	Documented string	Update	Limitations of record
PubMed/MEDLINE	Inception to 31 December 2022	("non-alcoholic fatty liver disease" OR NAFLD OR "fatty liver" OR "hepatic steatosis" OR "metabolic-associated fatty liver disease" OR MAFLD) AND ("type 2 diabetes" OR "type 2 diabetes mellitus" OR "diabetes mellitus" OR "incident diabetes" OR "new-onset diabetes") AND (cohort OR longitudinal OR "follow-up" OR prospective OR retrospective)	1 January 2023 to 22 July 2026; MASLD terms added; 1,187 records before deduplication	Exact original execution date, full database history, and database-specific original yield were not retained.
Scopus	Inception to 31 December 2022	TITLE-ABS-KEY(("non-alcoholic fatty liver disease" OR NAFLD OR "fatty liver" OR "hepatic steatosis" OR "metabolic-associated fatty liver disease" OR MAFLD) AND ("type 2 diabetes" OR "type 2 diabetes mellitus" OR "incident diabetes" OR "new-onset diabetes") AND (cohort OR longitudinal OR "follow-up" OR prospective OR retrospective))	1 January 2023 to 22 July 2026; MASLD terms added; 45 records before deduplication	Exact original execution date, full database history, and database-specific original yield were not retained.

Appendix 3 

**Table 6:** Mapping of reports arising from overlapping cohorts and shared data sources. This table identifies reports derived from the same or potentially overlapping cohorts, including NAGALA and the Kangbuk Samsung Health Study. These reports were treated as related analyses and were not interpreted as independent replications. NAGALA: Nonalcoholic Fatty Liver Disease in the Gifu Area, Longitudinal Analysis; ELSA-Brasil: Brazilian Longitudinal Study of Adult Health; MASLD: Metabolic Dysfunction-Associated Steatotic Liver Disease; UK: United Kingdom; NASH CRN: Nonalcoholic Steatohepatitis Clinical Research Network; CARDIA: Coronary Artery Risk Development in Young Adults; D2d: Vitamin D and Type 2 Diabetes Study; KoGES: Korean Genome and Epidemiology Study; HIV-NAT: Human Immunodeficiency Virus–Netherlands–Australia–Thailand Research Collaboration; TCLSIH: Tianjin Chronic Low-Grade Systemic Inflammation and Health; WRW: Wenzhou Real-World; CDARS: Clinical Data Analysis and Reporting System.

Dataset/cohort	Included reports	Handling
NAGALA/Murakami Memorial Hospital health-check data	[18], [47], [49], [52], [53], [63], [64], [66], [68], [69], [76], [77]; possibly related infrastructure for [36]	Treated as related reports; not interpreted as independent replications or pooled together.
Kangbuk/Samsung health-screening cohorts	[34], [41], [54], [59]; other Samsung reports assessed on a case-by-case basis	Treated as related where cohort identity or screening infrastructure overlapped; distinct clinical questions were retained.
Korean National Health Insurance Service young-adult cohorts	[20], [61], [62], [74]	Treated as related nationwide reports; differences in exposure definitions and study questions were retained.
ELSA-Brasil	[43], [58]	Treated as related reports addressing different steatotic liver disease definitions or analyses.
Taiwan health-check cohort	[60], [71]	Treated as related reports; severity and MASLD/cardiometabolic risk analyses were interpreted as distinct questions.
Other named cohorts	Busselton, UK Biobank, NASH CRN, CARDIA, D2d, KoGES, HIV-NAT, TCLSIH, Kailuan, WRW, and Hong Kong CDARS	No overlap was assumed unless cohort identity or recruitment information indicated shared participants.

Appendix 4 

**Table 7:** Study-level Newcastle-Ottawa Scale risk-of-bias assessment. The table presents Newcastle-Ottawa Scale assessments for all 61 included reports across the selection, comparability, and outcome domains. Based on review-specific thresholds, reports scoring 7-9 stars were classified as low risk, those scoring 5-6 stars as moderate risk, and those scoring fewer than 5 stars as high risk. NOS: Newcastle-Ottawa Scale.

Ref.	Study	Selection	Comparability	Outcome	Total NOS score	Overall judgment
17	Adams LA et al. (2009)	3/4	2/2	2/3	7/9	Low
18	Cai X et al. (2022)	2/4	2/2	1/3	5/9	Moderate
19	Cho HJ et al. (2019)	4/4	2/2	2/3	8/9	Low
20	Chung GE et al. (2021)	3/4	2/2	3/3	8/9	Low
21	Fan JG et al. (2007)	3/4	1/2	2/3	6/9	Moderate
22	Fukuda T et al. (2016)	4/4	2/2	2/3	8/9	Low
23	Kogiso T et al. (2021)	2/4	1/2	2/3	5/9	Moderate
24	Kim Y et al. (2022)	3/4	2/2	2/3	7/9	Low
25	Dai W et al. (2021)	3/4	2/2	2/3	7/9	Low
26	Tang X et al. (2022)	3/4	0/2	2/3	5/9	Moderate
27	Zhang H et al. (2022)	3/4	2/2	2/3	7/9	Low
28	Arase Y et al. (2009)	3/4	2/2	2/3	7/9	Low
29	Sanyal AJ et al. (2021)	3/4	1/2	2/3	6/9	Moderate
30	Li WD et al. (2015)	4/4	2/2	3/3	9/9	Low
31	Liu C et al. (2022)	3/4	2/2	2/3	7/9	Low
32	Nasr P et al. (2020)	2/4	2/2	2/3	6/9	Moderate
33	Xia Y et al. (2022)	4/4	2/2	2/3	8/9	Low
34	Bae JC et al. (2018)	4/4	2/2	2/3	8/9	Low
35	Tokita Y et al. (2017)	4/4	1/2	2/3	7/9	Low
36	Zheng X et al. (2021)	3/4	2/2	2/3	7/9	Low
37	Lee J et al. (2019)	3/4	2/2	2/3	7/9	Low
38	Sinn DH et al. (2019)	4/4	2/2	2/3	8/9	Low
39	Ming J et al. (2015)	4/4	2/2	3/3	9/9	Low
40	Lago-Sampedro A et al. (2024)	3/4	2/2	2/3	7/9	Low
41	Cho Y et al. (2023)	3/4	2/2	2/3	7/9	Low
42	Chen C et al. (2023)	3/4	2/2	2/3	7/9	Low
43	Lopes GW et al. (2024)	4/4	2/2	2/3	8/9	Low
44	Bae JH et al. (2025)	3/4	2/2	3/3	8/9	Low
45	Xie J et al. (2024)	3/4	2/2	2/3	7/9	Low
46	Corbin KD et al. (2023)	3/4	0/2	2/3	5/9	Moderate
47	Sakai K et al. (2024)	3/4	1/2	2/3	6/9	Moderate
48	Li N et al. (2023)	3/4	2/2	2/3	7/9	Low
49	Si R et al. (2024)	3/4	2/2	3/3	8/9	Low
50	Park SH et al. (2023)	3/4	2/2	2/3	7/9	Low
51	Han WM et al. (2023)	3/4	2/2	2/3	7/9	Low
52	Liang X et al. (2025)	3/4	2/2	2/3	7/9	Low
53	Xie X et al. (2023)	3/4	2/2	2/3	7/9	Low
54	Cho Y et al. (2023)	3/4	2/2	2/3	7/9	Low
55	Hatano Y et al. (2023)	4/4	2/2	2/3	8/9	Low

Appendix 5 

**Table 8:** Structured qualitative assessment of confidence in the evidence. The table summarizes confidence in the principal findings according to risk of bias, consistency, directness, precision, and potential publication or selective reporting bias. No pooled estimate was calculated, and no formal GRADE assessment was performed. GRADE: Grading of Recommendations Assessment, Development and Evaluation.

Major conclusion	Risk of bias	Consistency	Directness	Precision	Publication bias	Qualitative confidence	Reasoning
Overall direction of the association between steatotic liver disease and incident T2DM	Some concerns; lower risk of bias in adequately reported cohorts	Direction generally similar; magnitude variable	Moderate; exposure and outcome definitions vary	Often adequate in large cohorts but limited in small or selected studies	Possible; not statistically testable	Moderate for direction; lower for exact magnitude	Multiple longitudinal cohorts support a higher occurrence, but heterogeneous definitions and metabolic adjustment preclude a single quantitative estimate.
NAFLD in lean, non-overweight, or non-obese groups	Some concerns regarding subgroup selection and cohort representativeness	Generally positive, but definitions differ	Moderate	Variable; some estimates have wide intervals	Possible selective subgroup reporting	Low to moderate	Study-defined BMI groups are not interchangeable, and several estimates arise from single cohorts.
Persistent or newly developed NAFLD and lower risk after resolution or remission	Observational repeated-measures cohorts with concerns about attrition	Direction similar in several reports, but not universal	Moderate	Variable	Possible	Low to moderate	Improvement may co-occur with weight loss, dietary changes, physical activity, or changes in insulin sensitivity; reversibility is not established.
Greater steatosis, liver-fat, or fibrosis burden and incident T2DM	Selected biopsy and special clinical cohorts	Broad severity gradient, but dimensions differ	Limited by non-interchangeable measures	Variable	Possible	Low to moderate	Steatosis grade, quantitative liver fat, and fibrosis are related but represent different disease dimensions.
Subgroup, lifestyle, reproductive, genetic, and prediction findings	Frequently based on secondary analyses, small event numbers, data-driven thresholds, or studies without external validation	Rarely replicated independently	Variable	Often limited	High concern regarding positive or selectively reported findings	Low; hypothesis-generating	These findings should not be treated as evidence of established universal effect modification or individualized prediction.

Appendix 6 

**Table 9:** Structured effect-estimate matrix for the included reports. The table presents study-specific exposure definitions, comparator groups, adjusted effect measures, event counts, follow-up periods, overlap considerations, and the principal reasons why quantitative pooling was not performed across the 61 included reports. AIP: Atherogenic index of plasma; ALD: Alcohol-related liver disease; ALT: Alanine aminotransferase; APRI: Aspartate aminotransferase-to-platelet ratio index; ART: Antiretroviral therapy; CC: Cytosine-cytosine genotype; CDARS: Clinical Data Analysis and Reporting System; CMI: Cardiometabolic index; D2d: Vitamin D and Type 2 Diabetes Study; ELSA-Brasil: Brazilian Longitudinal Study of Adult Health; F0-F4: Fibrosis stages 0-4; FIB-4: Fibrosis-4 index; FLD: Fatty liver disease; FLI: Fatty liver index; GDM: Gestational diabetes mellitus; GGT: Gamma-glutamyl transferase; HbA1c: Glycated hemoglobin; HDL-C: High-density lipoprotein cholesterol; HSI: Hepatic steatosis index; KCNQ1: Potassium voltage-gated channel subfamily Q member 1 gene; LDL-C: Low-density lipoprotein cholesterol; MAFLD: Metabolic dysfunction-associated fatty liver disease; MASLD: Metabolic dysfunction-associated steatotic liver disease; MetALD: Metabolic dysfunction-associated steatotic liver disease with increased alcohol intake; NAGALA: Non-alcoholic Fatty Liver Disease in the Gifu Area, Longitudinal Analysis; NAFLD: Non-alcoholic fatty liver disease; NAFLD-LFS: Non-alcoholic fatty liver disease liver fat score; NASH: Non-alcoholic steatohepatitis; NFS: Non-alcoholic fatty liver disease fibrosis score; NHIS: National Health Insurance Service; SLD: Steatotic liver disease; T2DM: Type 2 diabetes mellitus; TCLSIH: Tianjin Chronic Low-Grade Systemic Inflammation and Health; TyG: Triglyceride-glucose index; TyG/HDL-C: Triglyceride-glucose index-to-high-density lipoprotein cholesterol ratio; TyG-WHtR: Triglyceride-glucose-waist-to-height ratio index; TyH-i: Triglyceride-glycated hemoglobin index; WHtR: Waist-to-height ratio; WRW: Wenzhou Real-World.

Ref.	Study	Evidence type	Exposure/comparison	Effect measure/reporting	Main adjusted estimate(s)	Events	Follow-up	Adjustment information	Potential quantitative group	Reason not pooled	Cohort overlap	Source URL
17	Adams LA et al. (2009)	Original report	ALT-defined NAFLD versus no NAFLD	Crude incidence/adjusted non-significance	Higher crude incidence; NAFLD was no longer statistically independent after adjustment (adjusted estimate not reported in the abstract)	20/106 (18.9%) versus 15/246 (6.1%) after excluding baseline diabetes	11.1 years	See full extraction; principal adjusted model prioritized where available	No	ALT surrogate exposure and no adjusted estimate reported	No overlap identified	PubMed
18	Cai X et al. (2022)	Original report	Five-predictor nomogram among participants with obesity and NAFLD	Prediction-model performance	Five predictors were selected from 17 variables; AUCs and the C-index indicated predictive performance, but numerical values were not reported in the abstract	Not reported in the PubMed abstract	Not reported in the PubMed abstract	See full extraction; principal adjusted model prioritized where available	No	Prediction model; no etiological comparator effect	NAGALA cohort; potentially overlaps with other NAGALA reports	PubMed
19	Cho HJ et al. (2019)	Original report	Persistent, resolved, incident, and no-NAFLD categories	Adjusted HRs for disease-status trajectories	Persistent NAFLD: HR 3.59 (95% CI 2.05-6.27); incident NAFLD: HR 1.94 (1.08-3.50); resolved NAFLD was not significantly different from no NAFLD	Not reported in the PubMed abstract	Serial follow-up; exact duration not reported in the PubMed abstract	See full extraction; principal adjusted model prioritized where available	No	Dynamic multicategory exposure	No overlap identified from the abstract	PubMed
20	Chung GE et al. (2021)	Original report	FLI-defined NAFLD versus control	Adjusted HR	Adjusted HR 4.97 (95% CI 4.90-5.05)	91,885 incident diabetes cases	Median 8.6 years	See full extraction; principal adjusted model prioritized where available	Potential FLI-defined group	Index-based exposure; claims-based outcome; distinct from imaging cohorts	No overlap identified	PubMed
21	Fan JG et al. (2007)	Original report	Fatty liver versus matched controls; obesity-stratified analyses	Incidence proportions	Incidence comparisons only; no adjusted association estimate reported in the abstract	Diabetes incidence: 20.3% versus 5.2%; non-obese subgroup: 11.1% versus 4.3%	Median 6 years	See full extraction; principal adjusted model prioritized where available	No	No adjusted effect estimate reported in the abstract	No overlap identified	PubMed
22	Fukuda T et al. (2016)	Original report	NAFLD and BMI strata; non-overweight defined as BMI <23 kg/m²	Adjusted HRs by BMI/NAFLD strata	Versus non-overweight without NAFLD: HR 3.59 (95% CI 2.14-5.76) for non-overweight NAFLD; HR 1.99 (1.47-2.69) for overweight without NAFLD; HR 6.77 (5.17-8.91) for overweight NAFLD	351 incident T2DM cases	Mean 12.8 years	See full extraction; principal adjusted model prioritized where available	Potential subgroup synthesis	Multiple BMI-defined comparator categories	No overlap identified	PubMed
23	Kogiso T et al. (2021)	Original report	Within-NAFLD clinical, obesity, fibrosis, and genetic risk factors	Within-NAFLD incidence/risk factors	Higher incidence among patients with obesity and the KCNQ1 CC genotype; numerical adjusted association not reported in the abstract	44 de novo diabetes cases; 10-year incidence of 17.9%	10-year incidence reported	See full extraction; principal adjusted model prioritized where available	No	No NAFLD-free comparator	No overlap identified	PubMed
24	Kim Y et al. (2022)	Original report	NAFLD versus no NAFLD, stratified by sex and menopausal status; severity assessed using serum fibrosis markers	Adjusted HRs by sex/menopausal status	Adjusted HR 4.63 (95% CI 4.17-5.14) in premenopausal women; 2.65 (2.02-3.48) in postmenopausal women; 2.16 (2.04-2.29) in men	8,381 incident T2D cases	Median 5.3 years	See full extraction; principal adjusted model prioritized where available	Potential subgroup synthesis	Sex-stratified estimates and prediction component	Cohort overlap not identifiable from the abstract	PubMed
25	Dai W et al. (2021)	Original report	Ultrasound-defined NAFLD versus no NAFLD in non-obese adults	Adjusted HR	Adjusted HR 2.3 (95% CI 1.7-3.2)	177 incident T2D cases	5 years	See full extraction; principal adjusted model prioritized where available	Potential ultrasound-defined non-obese group	Restricted non-obese population	No overlap identified	PubMed
26	Tang X et al. (2022)	Original report	Clinical outcomes among participants with NAFLD	Descriptive cumulative incidence	Descriptive outcome proportions; no adjusted NAFLD-versus-control diabetes estimate reported	Diabetes developed in 12.6% of male and 11.6% of female participants with NAFLD	11 years	See full extraction; principal adjusted model prioritized where available	No	No adjusted NAFLD-versus-control estimate	No overlap identified	PubMed
27	Zhang H et al. (2022)	Original report	FLI-defined NAFLD and sleep-pattern categories	Joint-exposure RR and interaction	NAFLD plus poor sleep pattern: RR 3.17 (95% CI 2.80-3.59); the overall sleep pattern did not significantly interact with NAFLD, but the interaction with insomnia was significant (P = 0.003)	8,774 incident T2DM cases	Median 11.0 years	See full extraction; principal adjusted model prioritized where available	No	Sleep-interaction study; main NAFLD effect not reported in the abstract	UK Biobank; no overlap with other included reports identified	PubMed
28	Arase Y et al. (2009)	Original report	Prediabetes, GGT, triglycerides, and physical activity within NAFLD	Adjusted HRs for within-NAFLD predictors	Prediabetes: HR 6.39 (95% CI 5.00-8.18); GGT >109 IU/L: HR 1.60 (1.22-2.02); triglycerides >150 mg/L: HR 1.28 (1.05-1.55); physical activity <60 min/week: HR 1.60 (1.25-2.00)	411 incident T2D cases	Mean 4.9 years	See full extraction; principal adjusted model prioritized where available	No	No NAFLD-free comparator	No overlap identified	PubMed
29	Sanyal AJ et al. (2021)	Original report	Histologic fibrosis stages F0-F2, F3, and F4	Incidence rates by fibrosis stage	Incidence-rate comparison; no T2DM-specific adjusted effect estimate reported in the abstract	T2DM incidence: 7.53 versus 4.45 events per 100 person-years for F4 versus F0-F2	Median 4 years	See full extraction; principal adjusted model prioritized where available	No	Histologic severity outcome; no adjusted T2DM effect	NASH Clinical Research Network	PubMed
30	Li WD et al. (2015)	Original report	NAFLD versus control and BMI categories	Adjusted RR	Adjusted RR 3.367 (95% CI 2.367-4.266) for NAFLD versus control	380 incident T2DM cases; cumulative incidence of 17.4% in NAFLD versus 4.1% in controls	Median 3.85 years (4-year follow-up; 17,223 person-years)	See full extraction; principal adjusted model prioritized where available	Potential ultrasound-defined general-population group	Different effect measure and diabetes definition	No overlap identified	PubMed
31	Liu C et al. (2022)	Original report	Current, former, and never-smoking categories among participants with NAFLD	Adjusted HR for smoking within NAFLD	Current versus never smoking: adjusted HR 2.05 (95% CI 1.43-2.94)	223/2,714 participants with NAFLD developed T2DM	16,446 person-years among participants with NAFLD	See full extraction; principal adjusted model prioritized where available	No	Risk modifier, not an NAFLD-versus-control comparison	No overlap identified	PubMed
32	Nasr P et al. (2020)	Original report	Steatosis grade, quantified liver-fat percentage, and change in liver fat	Adjusted HRs per steatosis or liver-fat unit	Adjusted HR 1.60 per steatosis grade; HR 1.03 per percentage-point increase in quantified liver fat; reduction in liver fat: HR 0.91 per percentage-point decrease	66/106 developed T2DM	Mean 23.2 years	See full extraction; principal adjusted model prioritized where available	No	Biopsy-based severity measures	No overlap identified	PubMed
33	Xia Y et al. (2022)	Original report	NAFLD versus no NAFLD, stratified by vegetable dietary-pattern intake	Adjusted HR and dietary interaction	NAFLD: HR 3.04 (95% CI 2.51-3.68); low vegetable intake: HR 4.08 (3.05-5.46); high vegetable intake: HR 2.38 (1.85-3.07); interaction P < 0.01	787 incident T2DM cases	93,873 person-years	See full extraction; principal adjusted model prioritized where available	Potential ultrasound-defined general-population group	Dietary interaction and cohort-specific adjustment	TCLSIH cohort	PubMed
34	Bae JC et al. (2018)	Original report	Sustained, intermittent, and never-NAFLD categories	Adjusted HRs for persistent/intermittent NAFLD	Sustained versus never NAFLD: HR 1.50 (95% CI 1.13-1.98); intermittent versus never NAFLD: HR 0.99 (0.76-1.31); sustained versus intermittent NAFLD: HR 1.50 (1.20-1.89)	Not reported in the PubMed abstract	Mean 4 years; annual assessments over 5 years	See full extraction; principal adjusted model prioritized where available	No	Time-varying/dynamic exposure and overlapping cohort	Kangbuk Samsung Health Study; overlaps with later Kangbuk Samsung reports	PubMed
35	Tokita Y et al. (2017)	Original report	Ultrasound-defined fatty liver versus no fatty liver, stratified by sex	Sex-specific relative risks	Relative risk 4.8 (95% CI 3.0-7.8) in men and 14.5 (7.0-30.1) in women	88 total cases: 29/232 versus 34/1,312 in men; 10/38 versus 15/826 in women	10 years	See full extraction; principal adjusted model prioritized where available	Potential subgroup synthesis	Sex-stratified estimates; diabetes criteria require verification	No overlap identified	PubMed
36	Zheng X et al. (2021)	Original report	Baseline NAFLD versus no NAFLD	Adjusted HR using propensity-score methods	Matched analysis: HR 1.98 (95% CI 1.41-2.80); multivariable matched-cohort analysis: HR 2.33 (1.63-3.32); propensity-score-adjusted analysis: HR 1.95 (1.39-2.75)	Not reported in the PubMed abstract	2004-2015 observation window; exact individual follow-up not reported in the abstract	See full extraction; principal adjusted model prioritized where available	Potential ultrasound-defined general-cohort group	Likely NAGALA overlap; diagnostic method and follow-up require verification	Likely Murakami/NAGALA data infrastructure; participant overlap requires verification in the full text	PubMed
37	Lee J et al. (2019)	Original report	NAFLD status and tertiles of waist-circumference change	Adjusted RR in participants with prediabetes	NAFLD: adjusted RR 1.81 (95% CI 1.47-2.21); waist-circumference-change tertiles: RR 1.64 (1.08-2.49), 1.73 (1.28-2.34), and 2.04 (1.42-2.93)	505 incident diabetes cases	Follow-up visits between 2008 and 2013; summary duration not stated in the abstract	See full extraction; principal adjusted model prioritized where available	No	Selected prediabetes population and waist-circumference-change modifier	Cohort overlap not identifiable from the abstract	PubMed
38	Sinn DH et al. (2019)	Original report	Lean NAFLD, overweight/obese without NAFLD, and overweight/obese with NAFLD versus lean without NAFLD	Adjusted HRs by lean/obesity and fibrosis-score strata	Versus lean without NAFLD: lean NAFLD, HR 1.18 (95% CI 1.03-1.35); overweight/obese without NAFLD, HR 1.06 (0.98-1.14); overweight/obese with NAFLD, HR 1.45 (1.34-1.57); lean NAFLD with intermediate/high NFS, HR 2.73 (2.10-3.55)	5,370 incident diabetes cases	Median 4.0 years; 236,446.6 person-years	See full extraction; principal adjusted model prioritized where available	Potential subgroup synthesis	Multiple strata; likely overlap with health-screening cohorts	Likely Korean health-screening dataset; possible Kangbuk Samsung overlap requires verification in the full text	PubMed
39	Ming J et al. (2015)	Original report	NAFLD versus no NAFLD	Adjusted RR	Adjusted RR for T2DM: 4.462 (95% CI 1.855-10.734); adjusted RR for prediabetes: 1.642 (0.965-2.793)	20 incident T2DM cases; 85 incident prediabetes cases	5 years	See full extraction; principal adjusted model prioritized where available	Potential ultrasound-defined general-population group	Small event count and wide confidence interval	No overlap identified	PubMed
40	Lago-Sampedro A et al. (2024)	Supplementary update	High versus low Mediterranean diet adherence within MAFLD	Adjusted estimate(s) as reported	Adjusted OR 0.52 (95% CI 0.31-0.87) for high versus low adherence; OR 0.35 (0.16-0.78) among participants without weight gain	98	7.5 years	Age, sex, abdominal obesity, weight gain, fasting glucose, family history, insulin resistance, hypertension, dyslipidemia, medications, and lifestyle variables	Secondary risk modifier	Different exposure, comparator, population, adjustment set, estimand, or overlapping cohort; see Methods	No overlap identified within the uploaded update set	Source
41	Cho Y et al. (2023)	Supplementary update	NAFLD only and NAFLD overlapping with MAFLD versus neither condition	Adjusted estimate(s) as reported	Women: HR 2.39 (95% CI 1.63-3.51) for NAFLD only and 5.75 (5.17-6.36) for MAFLD; men: HR 1.53 (1.25-1.88) and 2.60 (2.44-2.76), respectively	8,402	Median 5.5 years	Multivariable Cox models with time-dependent covariates; effect modification by sex assessed	Core association	Different exposure, comparator, population, adjustment set, estimand, or overlapping cohort; see Methods	Kangbuk Samsung cohort; interpret alongside the other Cho Y et al. (2023) report	Source
42	Chen C et al. (2023)	Supplementary update	Consistent non-NAFLD, incident NAFLD, NAFLD remission, and sustained NAFLD	Adjusted estimate(s) as reported	Incident NAFLD versus consistent non-NAFLD: OR 1.43 (95% CI 1.10-1.86); remission versus sustained NAFLD: OR 0.48 (0.29-0.80)	484	Median 3.5 years	Multivariable models including obesity measures and changes in BMI or waist circumference	Core association and disease trajectory	Different exposure, comparator, population, adjustment set, estimand, or overlapping cohort; see Methods	No overlap identified within the uploaded update set	Source
43	Lopes GW et al. (2024)	Supplementary update	NAFLD and MASLD versus absence of the respective condition	Adjusted estimate(s) as reported	Adjusted HR 1.78 (95% CI 1.58-2.01) for NAFLD and 1.88 (1.67-2.12) for MASLD	1,157	Median 9.43 years	Multivariable Cox models; interaction by race or skin color examined	Core association and definition comparison	Different exposure, comparator, population, adjustment set, estimand, or overlapping cohort; see Methods	No overlap identified within the uploaded update set	Source
44	Bae JH et al. (2025)	Supplementary update	Fatty liver index categories: low, <30; intermediate, 30-59; and high, ≥60	Adjusted estimate(s) as reported	High versus low FLI: HR 3.42 (95% CI 2.91-4.00); after adjustment for insulin sensitivity and secretion markers: HR 1.96 (1.54-2.50)	1,006	Median 12.4 years; observation for up to 18 years	Age, sex, blood pressure, BMI, cholesterol measures, insulin sensitivity, and secretion markers	Core non-invasive-marker evidence	Different exposure, comparator, population, adjustment set, estimand, or overlapping cohort; see Methods	KoGES cohort	Source
45	Xie J et al. (2024)	Supplementary update	Smoking cessation and post-cessation weight gain, stratified by NAFLD status	Adjusted estimate(s) as reported	Among participants with NAFLD, HRs versus current smokers were 0.41, 2.39, 4.48, and 6.42 across increasing post-cessation weight-gain categories	363	7 years; 39,259 person-years	Age, BMI, drinking, liver enzymes, lipids, creatinine, albumin, and systolic blood pressure	Secondary lifestyle and weight modifier	Different exposure, comparator, population, adjustment set, estimand, or overlapping cohort; see Methods	No overlap identified within the uploaded update set	Source
46	Corbin KD et al. (2023)	Supplementary update	HSI, NAFLD-LFS, FIB-4, and APRI at baseline	Adjusted estimate(s) as reported	NAFLD-LFS: HR 1.35 (95% CI 1.07-1.70); APRI: HR 2.36 (1.23-4.54); vitamin D did not modify the associations	Not reported in the extracted abstract	Median 2.5 years	Longitudinal multivariable analysis within the parent randomized trial	Core non-invasive-marker evidence	Different exposure, comparator, population, adjustment set, estimand, or overlapping cohort; see Methods	D2d trial	Source
47	Sakai K et al. (2024)	Supplementary update	NAFLD and MASLD classifications	Adjusted estimate(s) as reported	Adjusted OR 6.00 (95% CI 4.84-7.45) for NAFLD and 6.58 (5.29-8.17) for MASLD; AUC 0.799 versus 0.807	NAFLD: 230/2,785; MASLD: 234/2,682	5 years	Age, sex, smoking status, and exercise habits	Core association and definition comparison	Different exposure, comparator, population, adjustment set, estimand, or overlapping cohort; see Methods	NAGALA cohort; overlaps with the Si, Liang, and Xie reports	Source
48	Li N et al. (2023)	Supplementary update	Lean NAFLD and overweight or obese NAFLD versus lean participants without NAFLD	Adjusted estimate(s) as reported	Lean NAFLD versus lean non-NAFLD: adjusted HR 3.83 (95% CI 2.02-7.24)	Not clearly reported in the extracted abstract	2 years	Multivariable Cox regression including metabolic and anthropometric factors	Core lean-NAFLD subgroup evidence	Different exposure, comparator, population, adjustment set, estimand, or overlapping cohort; see Methods	No overlap identified within the uploaded update set	Source
49	Si R et al. (2024)	Supplementary update	Hepatic steatosis index quartiles	Adjusted estimate(s) as reported	Compared with Q1, adjusted HRs were 1.09 (95% CI 0.61-1.93), 1.16 (0.68-1.98), and 3.30 (2.04-5.33) for Q2, Q3, and Q4, respectively	238	Mean 6.1 years	Age, alcohol consumption, smoking, systolic and diastolic blood pressure, triglycerides, and total cholesterol	Core non-invasive-marker evidence	Different exposure, comparator, population, adjustment set, estimand, or overlapping cohort; see Methods	NAGALA cohort; overlaps with the Sakai, Liang, and Xie X reports	Source
50	Park SH et al. (2023)	Supplementary update	NAFLD only, MAFLD only, both definitions, and non-fatty-liver metabolic-dysfunction groups	Adjusted estimate(s) as reported	Versus non-FLD without metabolic dysfunction: HR 2.68 (95% CI 1.58-4.53) for NAFLD only, 6.17 (4.96-7.68) for both definitions, and 8.30 (6.13-11.24) for MAFLD only	1,296	Median 5.0 years	Fully adjusted Cox regression including metabolic, hepatic, renal, and lifestyle covariates	Core association and definition comparison	Different exposure, comparator, population, adjustment set, estimand, or overlapping cohort; see Methods	Samsung Medical Center cohort; distinct from the Kangbuk Samsung reports unless subsequent cohort-level verification indicates otherwise	Source
51	Han WM et al. (2023)	Supplementary update	Baseline NAFLD and NAFLD plus NASH with significant activity and fibrosis	Adjusted estimate(s) as reported	NAFLD: adjusted HR 2.83 (95% CI 1.26-6.36); NAFLD plus NASH: adjusted HR 3.12 (1.05-9.26)	Incidence of 10.9 per 1,000 person-years	2013-2022; 2,552 person-years	Age, sex, family history, ART duration and exposures, smoking, statin use, time-updated BMI, hypertension, and dyslipidemia	Core special-population evidence	Different exposure, comparator, population, adjustment set, estimand, or overlapping cohort; see Methods	HIV-NAT cohort	Source
52	Liang X et al. (2025)	Supplementary update	Baseline triglyceride-glucose index	Adjusted estimate(s) as reported	U-shaped association with an inflection point at TyG 7.82; below the threshold: HR 0.07 (95% CI 0.01-0.32); above the threshold: HR 1.70 (1.19-2.44) per unit	203	Up to 11 years	Cox models with clinically and statistically selected metabolic confounders	Secondary biomarker within NAFLD	Different exposure, comparator, population, adjustment set, estimand, or overlapping cohort; see Methods	NAGALA cohort; overlaps with the Sakai, Si, and Xie X reports	Source
53	Xie X et al. (2023)	Supplementary update	Baseline fasting triglyceride concentration	Adjusted estimate(s) as reported	U-shaped association; below a triglyceride level of 53 mg/dL: HR 0.413 (95% CI 0.220-0.778) per 10 mg/dL; above the threshold: HR 1.091 (1.046-1.137)	39	Median 6.05 years	Fully adjusted Cox and two-piecewise regression models	Secondary biomarker within NAFLD	Different exposure, comparator, population, adjustment set, estimand, or overlapping cohort; see Methods	NAGALA cohort; overlaps with the Sakai, Si, and Liang reports	Source
54	Cho Y et al. (2023)	Supplementary update	Prior gestational diabetes and ultrasound-diagnosed NAFLD, separately and jointly	Adjusted estimate(s) as reported	HR 2.61 (95% CI 2.06-3.31) for prior GDM alone, 2.26 (1.96-2.59) for NAFLD alone, and 6.45 (5.19-8.00) for both	1,515	Median 3.9 years	Insulin resistance, BMI, and other potential confounders included as time-dependent covariates	Secondary high-risk subgroup and interaction	Different exposure, comparator, population, adjustment set, estimand, or overlapping cohort; see Methods	Kangbuk Samsung cohort; interpret alongside the other Cho Y et al. (2023) NAFLD-only report	Source
55	Hatano Y et al. (2023)	Supplementary update	CT-defined NAFLD versus no NAFLD, stratified by race	Adjusted estimate(s) as reported	Adjusted risk difference of 4.1% (95% CI 0.3%-7.9%) in White participants and −1.9% (−5.7% to 2.0%) in Black participants	3.7% of White and 8.0% of Black participants developed T2DM	Approximately 5 years	Targeted maximum-likelihood estimation with machine-learning adjustment for a comprehensive set of confounders	Core association and racial-subgroup evidence	Different exposure, comparator, population, adjustment set, estimand, or overlapping cohort; see Methods	CARDIA cohort	Source
56	Anastasiou G et al. (2023)	Corrected supplementary update	FLI-defined probable NAFLD versus lower FLI	Adjusted HR	HR 3.14 (95% CI 1.50-6.59) for FLI ≥60 versus <60	Approximately 11% of 882 participants	Median follow-up of approximately 6 years (IQR, 4-10 years); probable NAFLD defined as FLI ≥60	Principal adjusted analysis prioritized; metabolic indices interpreted cautiously	No pooled estimate	Clinical or methodological heterogeneity, cohort overlap, or both	Independent Greek clinical cohort	DOI 10.3390/diagnostics13030503
57	Petroni ML et al. (2023)	Corrected supplementary update	Within-NAFLD lifestyle/weight-change analysis	Adjusted OR	Percentage weight change: OR 0.57 (95% CI 0.41-0.79)	48 new cases among 363 participants without baseline diabetes	Up to 60 months, with visits every 6-12 months; established NAFLD at enrollment	Principal adjusted analysis prioritized; metabolic indices interpreted cautiously	No pooled estimate	Clinical or methodological heterogeneity, cohort overlap, or both	Independent Italian clinical cohorts	DOI 10.3390/nu15030792
58	Faria LC et al. (2023)	Corrected supplementary update	Liver steatosis versus no steatosis	Adjusted longitudinal association	Higher risk of incident diabetes with baseline steatosis	Reported in the source	Prospective follow-up; liver steatosis assessed at baseline using a validated steatosis assessment in ELSA-Brasil	Principal adjusted analysis prioritized; metabolic indices interpreted cautiously	No pooled estimate	Clinical or methodological heterogeneity, cohort overlap, or both	ELSA-Brasil; overlaps with [43]	DOI 10.1590/0102-311XEN090522
59	Sinn DH et al. (2023)	Corrected supplementary update	Regressed versus persistent ultrasound-defined NAFLD	Adjusted HR	HR 0.81 (95% CI 0.72-0.92)	1,768	NAFLD reassessed after 1-2 years; median diabetes follow-up of 4 years; abdominal ultrasonography	Principal adjusted analysis prioritized; metabolic indices interpreted cautiously	No pooled estimate	Clinical or methodological heterogeneity, cohort overlap, or both	Samsung/Kangbuk health-screening infrastructure; related to [34], [41], and [54]	DOI 10.1371/journal.pone.0288820
60	Shih CI et al. (2024)	Corrected supplementary update	Ultrasound-defined fatty-liver severity categories	Adjusted HRs	Mild fatty liver: HR 3.22 (95% CI 2.56-4.07); moderate/severe fatty liver: HR 5.88 (4.44-7.81)	Reported in the source	Mean diabetes follow-up of approximately 6.75 years; fatty-liver severity assessed using ultrasonography	Principal adjusted analysis prioritized; metabolic indices interpreted cautiously	No pooled estimate	Clinical or methodological heterogeneity, cohort overlap, or both	Taiwan health-check cohort; related to [71]	DOI 10.1007/s12072-023-10576-z
61	Park KY et al. (2024)	Corrected supplementary update	Dynamic FLI-defined NAFLD status	Adjusted HR	Persistent NAFLD versus persistent non-NAFLD: HR 5.38	26,464	Median 8.3 years; two health examinations; NAFLD defined as FLI ≥60, with status changes classified	Principal adjusted analysis prioritized; metabolic indices interpreted cautiously	No pooled estimate	Clinical or methodological heterogeneity, cohort overlap, or both	Korean NHIS young-adult dataset; related to [20], [62], and [74]	DOI 10.1016/j.amepre.2023.11.021
62	Ha J et al. (2024)	Corrected supplementary update	MAFLD versus non-MAFLD and overlapping NAFLD categories	Adjusted HR	MAFLD: HR 6.148 (95% CI 6.084-6.212)	182,291	Median 9.5 years; FLI and metabolic criteria used to classify MAFLD/NAFLD groups	Principal adjusted analysis prioritized; metabolic indices interpreted cautiously	No pooled estimate	Clinical or methodological heterogeneity, cohort overlap, or both	Korean NHIS young-adult dataset; related to [20], [61], and [74]	DOI 10.1016/j.diabres.2024.111584
63	Wu C et al. (2024)	Corrected supplementary update	ZJU index quartiles	Adjusted HR	Q4 versus Q1: HR 2.519 (95% CI 1.297-4.891)	373	93,350 person-years; median 5.9 years; ZJU index used as a surrogate NAFLD-related metabolic index	Principal adjusted analysis prioritized; metabolic indices interpreted cautiously	No pooled estimate	Clinical or methodological heterogeneity, cohort overlap, or both	NAGALA; overlaps with multiple NAGALA reports	DOI 10.2147/DMSO.S446042
64	Wang Y et al. (2025)	Corrected supplementary update	Prediction model including ultrasound-defined fatty liver	Prediction-model association/performance	Fatty liver was retained as a predictive feature; model AUC of approximately 0.90	373 in the source cohort	Approximately 8-year analytical horizon; ultrasound-defined fatty liver and clinical/metabolic predictors	Principal adjusted analysis prioritized; metabolic indices interpreted cautiously	No pooled estimate	Clinical or methodological heterogeneity, cohort overlap, or both	NAGALA	DOI 10.3389/fendo.2025.1465032
65	Chen Y et al. (2025)	Corrected supplementary update	AIP tertiles within NAFLD	Adjusted OR	Q3 versus Q1: OR 1.99 (95% CI 1.29-3.08); continuous OR 2.76 (1.54-4.93)	Reported in the source	Longitudinal follow-up; NAFLD present at baseline; AIP evaluated as a within-NAFLD risk marker	Principal adjusted analysis prioritized; metabolic indices interpreted cautiously	No pooled estimate	Clinical or methodological heterogeneity, cohort overlap, or both	Japanese secondary cohort; possibly derived from the NAGALA dataset	DOI 10.1186/s12902-025-02046-4
66	Yang L et al. (2025)	Corrected supplementary update	Remnant-cholesterol categories within NAFLD	Adjusted HR	The highest category showed an increased risk of T2DM; adjusted HR approximately 1.68 in the reported analysis	233	Longitudinal health-check follow-up; ultrasound-defined NAFLD; remnant cholesterol evaluated	Principal adjusted analysis prioritized; metabolic indices interpreted cautiously	No pooled estimate	Clinical or methodological heterogeneity, cohort overlap, or both	NAGALA-related Japanese health-check data	DOI 10.1186/s13098-025-01828-z
67	Sun T and Liu J (2025)	Corrected supplementary update	TyG and TyG/HDL-C within NAFLD	Adjusted ORs	TyG: OR 1.96 (95% CI 1.67-2.30); TyG/HDL-C: OR 1.90 (1.60-2.26)	Reported in the source	3 years; TyG and TyG/HDL-C evaluated as metabolic risk markers in a NAFLD cohort	Principal adjusted analysis prioritized; metabolic indices interpreted cautiously	No pooled estimate	Clinical or methodological heterogeneity, cohort overlap, or both	No established overlap	DOI 10.3389/fendo.2025.1594548
68	Cao C et al. (2025)	Corrected supplementary update	TyG within MASLD	Adjusted HR	HR 1.48 (95% CI 1.05-2.09); nonlinear threshold of 7.94	204	Mean 6.0 years; baseline MASLD cohort; TyG index evaluated	Principal adjusted analysis prioritized; metabolic indices interpreted cautiously	No pooled estimate	Clinical or methodological heterogeneity, cohort overlap, or both	NAGALA-related Japanese cohort	DOI 10.3389/fendo.2025.1516187
69	Hou S et al. (2025)	Corrected supplementary update	CMI quartiles within NAFLD	Adjusted OR	Q4 versus Q1: OR 3.239 (95% CI 1.993-5.264)	204	Longitudinal follow-up; NAFLD present at baseline; cardiometabolic index (CMI) evaluated	Principal adjusted analysis prioritized; metabolic indices interpreted cautiously	No pooled estimate	Clinical or methodological heterogeneity, cohort overlap, or both	NAGALA-related Japanese cohort	DOI 10.1186/s12902-024-01828-6
70	Choque Vargas C et al. (2025)	Corrected supplementary update	Advanced versus lower fibrosis in biopsy-proven MASLD	Adjusted HR	Advanced fibrosis: HR approximately 3.04	Reported in the source	Approximately 10 years of historical follow-up; basal fibrosis categorized using liver biopsy	Principal adjusted analysis prioritized; metabolic indices interpreted cautiously	No pooled estimate	Clinical or methodological heterogeneity, cohort overlap, or both	Independent biopsy cohort	DOI 10.1097/MEG.0000000000002943
71	Shih CI et al. (2025)	Corrected supplementary update	MASLD versus non-SLD; cardiometabolic-risk strata	Incidence/risk estimates	Higher incident diabetes rates in MASLD, graded by risk-factor burden	Reported in the source	Longitudinal follow-up using health examinations; MASLD versus non-SLD classified according to steatosis and cardiometabolic risk factors	Principal adjusted analysis prioritized; metabolic indices interpreted cautiously	No pooled estimate	Clinical or methodological heterogeneity, cohort overlap, or both	Taiwan health-check cohort; related to [60]	DOI 10.1002/kjm2.70077
72	Hou C et al. (2025)	Corrected supplementary update	Transition from NAFLD to T2DM according to insulin-resistance burden	Adjusted HR	Transition to T2DM: HR 1.41 (95% CI 1.11-1.78)	A total of 12.65% of the incident-NAFLD subgroup progressed to T2DM in the reported analysis	Median 13.6 years; incident NAFLD followed by transitions to cardiovascular-kidney-metabolic outcomes	Principal adjusted analysis prioritized; metabolic indices interpreted cautiously	No pooled estimate	Clinical or methodological heterogeneity, cohort overlap, or both	UK Biobank	DOI 10.1186/s12933-025-02953-9
73	Zhou XD et al. (2026)	Corrected supplementary update	Data-driven MASLD clusters	Adjusted HRs	Cardiometabolic cluster: HR 3.418 in WRW and 2.761 in Hong Kong CDARS	Reported by cohort	Longitudinal cohort follow-up; MASLD clusters defined using age, BMI, HbA1c, ALT, LDL-C, and triglycerides	Principal adjusted analysis prioritized; metabolic indices interpreted cautiously	No pooled estimate	Clinical or methodological heterogeneity, cohort overlap, or both	Multiple Asian datasets; not independent of underlying source cohorts used for other outcomes	DOI 10.1016/j.jhepr.2025.101645
74	Chung GE et al. (2026)	Corrected supplementary update	SLD subtype categories versus non-SLD	Adjusted HRs	Higher incidence of T2DM across MASLD, MetALD, and ALD subtypes	72,028	Median follow-up of approximately 10.6 years; FLI-based steatotic liver disease subtypes classified using cardiometabolic and alcohol criteria	Principal adjusted analysis prioritized; metabolic indices interpreted cautiously	No pooled estimate	Clinical or methodological heterogeneity, cohort overlap, or both	Korean NHIS young-adult dataset; related to [20], [61], and [62]	DOI 10.1111/dom.70381
75	Huo Z et al. (2026)	Corrected supplementary update	Incident MASLD versus matched non-MASLD, stratified by age at onset	Adjusted HRs	Onset before 40 years: HR 6.18 (95% CI 4.28-8.93); association attenuated with older age at onset	2,301	Median 11.8 years; age at MASLD onset identified using serial health examinations	Principal adjusted analysis prioritized; metabolic indices interpreted cautiously	No pooled estimate	Clinical or methodological heterogeneity, cohort overlap, or both	Kailuan cohort	DOI 10.1016/j.cgh.2026.06.028
76	Chen N et al. (2026)	Corrected supplementary update	TyG-WHtR and other indices within NAFLD	Adjusted association/prediction performance	TyG-WHtR was independently associated with incident T2DM and had the highest discrimination among the indices tested	Reported in the source	12-year prospective cohort analysis; metabolic composite indices, including TyG-WHtR, assessed	Principal adjusted analysis prioritized; metabolic indices interpreted cautiously	No pooled estimate	Clinical or methodological heterogeneity, cohort overlap, or both	Secondary NAFLD cohort; possible shared Japanese dataset should be interpreted cautiously	DOI 10.3389/fendo.2026.1805902
77	Wei C et al. (2026)	Corrected supplementary update	TyH-i within NAFLD	Adjusted association/prediction performance	Higher TyH-i was associated with an increased risk of incident T2DM	223	Median 5.21 years; NAFLD present at baseline; triglyceride-glycated hemoglobin index (TyH-i) evaluated	Principal adjusted analysis prioritized; metabolic indices interpreted cautiously	No pooled estimate	Clinical or methodological heterogeneity, cohort overlap, or both	NAGALA-related Japanese cohort	DOI 10.1371/journal.pone.0350633

Appendix 7 

**Table 10:** Full-text exclusions from the corrected supplementary search. This table lists the 12 full-text reports excluded during the corrected supplementary eligibility assessment and provides one primary exclusion reason for each report. FHTHWA: Fasting plasma glucose-high-density lipoprotein cholesterol-triglycerides-hemoglobin A1c-waist circumference-age index; KoGES-HEXA: Korean Genome and Epidemiology Study-Health Examinees; MASLD: Metabolic dysfunction-associated steatotic liver disease; NAGALA: Nonalcoholic Fatty Liver Disease in the Gifu Area, Longitudinal Analysis; NAFLD: Non-alcoholic fatty liver disease; PCOS: Polycystic ovary syndrome; T2DM: Type 2 diabetes mellitus.

Report	Year	Primary exclusion reason
The product of waist-to-height ratio and glycated hemoglobin, a novel predictor of diabetes in East Asian populations: insights from two large East Asian cohort studies	2025	General diabetes-prediction model; no steatotic liver disease exposure of interest.
Metabolic-associated fatty liver disease is characterized by post-oral-glucose-load hyperinsulinemia in individuals with mild metabolic alterations	2024	Cross-sectional metabolic physiology study; no longitudinal assessment of incident T2DM.
Associations of very low lipoprotein(a) levels with risks of new-onset diabetes and non-alcoholic liver disease	2024	Ineligible exposure: lipoprotein(a) was the primary exposure, while diabetes and fatty liver were separate outcomes.
Metabolic dysfunction-associated steatotic liver disease and new-onset diabetes mellitus after liver transplantation	2025	Post-transplant population; the temporal ordering of MASLD before new-onset diabetes was not adequately established for the review question.
Diagnosis of hepatic steatosis and steatohepatitis in people with new-onset type 2 diabetes: a multidisciplinary approach	2024	Reverse temporal direction: hepatic steatosis was assessed in people with new-onset T2DM.
Impact of body mass index on disease progression and outcomes in patients with nonalcoholic fatty liver disease	2023	Focused on NAFLD progression and clinical outcomes without incident T2DM as an eligible longitudinal endpoint.
The first human normative ranges and biomarker performance of dimethylguanidino valeric acid isoforms in fatty liver disease	2024	Biomarker study in fatty liver disease without an incident T2DM outcome.
Oxidative balance score and new-onset type 2 diabetes mellitus in Korean adults without non-alcoholic fatty liver disease: Korean Genome and Epidemiology Study–Health Examinees (KoGES-HEXA) cohort	2024	Population explicitly restricted to participants without NAFLD; no steatotic liver disease exposure comparison.
Alpha-lipoic acid administration improved both peripheral sensitivity to insulin and liver clearance of insulin, reducing the potential risk of diabetes and nonalcoholic fatty liver disease in overweight/obese patients with PCOS	2024	Intervention study in patients with PCOS; no longitudinal incident T2DM outcome linked to baseline steatotic liver disease.
Use of the FHTHWA index as a novel approach for predicting the incidence of diabetes in a Japanese population without diabetes: data analysis study	2025	General diabetes-prediction index; no steatotic liver disease exposure of interest.
Relationship between liver fat, pancreatic fat, and new-onset type 2 diabetes mellitus in patients with metabolic dysfunction-associated fatty liver disease	2025	Case-control or cross-sectional comparison of participants with new-onset T2DM and controls rather than a baseline diabetes-free longitudinal cohort.
Assessing the usefulness of a newly proposed metabolic score for visceral fat in predicting future diabetes: results from the NAGALA cohort study	2023	General visceral-fat score used as a diabetes predictor; liver disease was not the exposure of interest.

Appendix 8 

Appendix 9

Appendix 10

Appendix 11
